# Search for single top-quark production via flavour-changing neutral currents at 8 TeV with the ATLAS detector

**DOI:** 10.1140/epjc/s10052-016-3876-4

**Published:** 2016-01-29

**Authors:** G. Aad, B. Abbott, J. Abdallah, O. Abdinov, R. Aben, M. Abolins, O. S. AbouZeid, H. Abramowicz, H. Abreu, R. Abreu, Y. Abulaiti, B. S. Acharya, L. Adamczyk, D. L. Adams, J. Adelman, S. Adomeit, T. Adye, A. A. Affolder, T. Agatonovic-Jovin, J. Agricola, J. A. Aguilar-Saavedra, S. P. Ahlen, F. Ahmadov, G. Aielli, H. Akerstedt, T. P. A. Åkesson, A. V. Akimov, G. L. Alberghi, J. Albert, S. Albrand, M. J. Alconada Verzini, M. Aleksa, I. N. Aleksandrov, C. Alexa, G. Alexander, T. Alexopoulos, M. Alhroob, G. Alimonti, L. Alio, J. Alison, S. P. Alkire, B. M. M. Allbrooke, P. P. Allport, A. Aloisio, A. Alonso, F. Alonso, C. Alpigiani, A. Altheimer, B. Alvarez Gonzalez, D. Álvarez Piqueras, M. G. Alviggi, B. T. Amadio, K. Amako, Y. Amaral Coutinho, C. Amelung, D. Amidei, S. P. Amor Dos Santos, A. Amorim, S. Amoroso, N. Amram, G. Amundsen, C. Anastopoulos, L. S. Ancu, N. Andari, T. Andeen, C. F. Anders, G. Anders, J. K. Anders, K. J. Anderson, A. Andreazza, V. Andrei, S. Angelidakis, I. Angelozzi, P. Anger, A. Angerami, F. Anghinolfi, A. V. Anisenkov, N. Anjos, A. Annovi, M. Antonelli, A. Antonov, J. Antos, F. Anulli, M. Aoki, L. Aperio Bella, G. Arabidze, Y. Arai, J. P. Araque, A. T. H. Arce, F. A. Arduh, J-F. Arguin, S. Argyropoulos, M. Arik, A. J. Armbruster, O. Arnaez, V. Arnal, H. Arnold, M. Arratia, O. Arslan, A. Artamonov, G. Artoni, S. Asai, N. Asbah, A. Ashkenazi, B. Åsman, L. Asquith, K. Assamagan, R. Astalos, M. Atkinson, N. B. Atlay, K. Augsten, M. Aurousseau, G. Avolio, B. Axen, M. K. Ayoub, G. Azuelos, M. A. Baak, A. E. Baas, M. J. Baca, C. Bacci, H. Bachacou, K. Bachas, M. Backes, M. Backhaus, P. Bagiacchi, P. Bagnaia, Y. Bai, T. Bain, J. T. Baines, O. K. Baker, E. M. Baldin, P. Balek, T. Balestri, F. Balli, E. Banas, Sw. Banerjee, A. A. E. Bannoura, H. S. Bansil, L. Barak, E. L. Barberio, D. Barberis, M. Barbero, T. Barillari, M. Barisonzi, T. Barklow, N. Barlow, S. L. Barnes, B. M. Barnett, R. M. Barnett, Z. Barnovska, A. Baroncelli, G. Barone, A. J. Barr, F. Barreiro, J. Barreiro Guimarães da Costa, R. Bartoldus, A. E. Barton, P. Bartos, A. Basalaev, A. Bassalat, A. Basye, R. L. Bates, S. J. Batista, J. R. Batley, M. Battaglia, M. Bauce, F. Bauer, H. S. Bawa, J. B. Beacham, M. D. Beattie, T. Beau, P. H. Beauchemin, R. Beccherle, P. Bechtle, H. P. Beck, K. Becker, M. Becker, M. Beckingham, C. Becot, A. J. Beddall, A. Beddall, V. A. Bednyakov, C. P. Bee, L. J. Beemster, T. A. Beermann, M. Begel, J. K. Behr, C. Belanger-Champagne, W. H. Bell, G. Bella, L. Bellagamba, A. Bellerive, M. Bellomo, K. Belotskiy, O. Beltramello, O. Benary, D. Benchekroun, M. Bender, K. Bendtz, N. Benekos, Y. Benhammou, E. Benhar Noccioli, J. A. Benitez Garcia, D. P. Benjamin, J. R. Bensinger, S. Bentvelsen, L. Beresford, M. Beretta, D. Berge, E. Bergeaas Kuutmann, N. Berger, F. Berghaus, J. Beringer, C. Bernard, N. R. Bernard, C. Bernius, F. U. Bernlochner, T. Berry, P. Berta, C. Bertella, G. Bertoli, F. Bertolucci, C. Bertsche, D. Bertsche, M. I. Besana, G. J. Besjes, O. Bessidskaia Bylund, M. Bessner, N. Besson, C. Betancourt, S. Bethke, A. J. Bevan, W. Bhimji, R. M. Bianchi, L. Bianchini, M. Bianco, O. Biebel, D. Biedermann, S. P. Bieniek, M. Biglietti, J. Bilbao De Mendizabal, H. Bilokon, M. Bindi, S. Binet, A. Bingul, C. Bini, S. Biondi, C. W. Black, J. E. Black, K. M. Black, D. Blackburn, R. E. Blair, J.-B. Blanchard, J. E. Blanco, T. Blazek, I. Bloch, C. Blocker, W. Blum, U. Blumenschein, G. J. Bobbink, V. S. Bobrovnikov, S. S. Bocchetta, A. Bocci, C. Bock, M. Boehler, J. A. Bogaerts, D. Bogavac, A. G. Bogdanchikov, C. Bohm, V. Boisvert, T. Bold, V. Boldea, A. S. Boldyrev, M. Bomben, M. Bona, M. Boonekamp, A. Borisov, G. Borissov, S. Borroni, J. Bortfeldt, V. Bortolotto, K. Bos, D. Boscherini, M. Bosman, J. Boudreau, J. Bouffard, E. V. Bouhova-Thacker, D. Boumediene, C. Bourdarios, N. Bousson, A. Boveia, J. Boyd, I. R. Boyko, I. Bozic, J. Bracinik, A. Brandt, G. Brandt, O. Brandt, U. Bratzler, B. Brau, J. E. Brau, H. M. Braun, S. F. Brazzale, W. D. Breaden Madden, K. Brendlinger, A. J. Brennan, L. Brenner, R. Brenner, S. Bressler, K. Bristow, T. M. Bristow, D. Britton, D. Britzger, F. M. Brochu, I. Brock, R. Brock, J. Bronner, G. Brooijmans, T. Brooks, W. K. Brooks, J. Brosamer, E. Brost, J. Brown, P. A. Bruckman de Renstrom, D. Bruncko, R. Bruneliere, A. Bruni, G. Bruni, M. Bruschi, N. Bruscino, L. Bryngemark, T. Buanes, Q. Buat, P. Buchholz, A. G. Buckley, S. I. Buda, I. A. Budagov, F. Buehrer, L. Bugge, M. K. Bugge, O. Bulekov, D. Bullock, H. Burckhart, S. Burdin, C. D. Burgard, B. Burghgrave, S. Burke, I. Burmeister, E. Busato, D. Büscher, V. Büscher, P. Bussey, J. M. Butler, A. I. Butt, C. M. Buttar, J. M. Butterworth, P. Butti, W. Buttinger, A. Buzatu, A. R. Buzykaev, S. Cabrera Urbán, D. Caforio, V. M. Cairo, O. Cakir, N. Calace, P. Calafiura, A. Calandri, G. Calderini, P. Calfayan, L. P. Caloba, D. Calvet, S. Calvet, R. Camacho Toro, S. Camarda, P. Camarri, D. Cameron, R. Caminal Armadans, S. Campana, M. Campanelli, A. Campoverde, V. Canale, A. Canepa, M. Cano Bret, J. Cantero, R. Cantrill, T. Cao, M. D. M. Capeans Garrido, I. Caprini, M. Caprini, M. Capua, R. Caputo, R. Cardarelli, F. Cardillo, T. Carli, G. Carlino, L. Carminati, S. Caron, E. Carquin, G. D. Carrillo-Montoya, J. R. Carter, J. Carvalho, D. Casadei, M. P. Casado, M. Casolino, E. Castaneda-Miranda, A. Castelli, V. Castillo Gimenez, N. F. Castro, P. Catastini, A. Catinaccio, J. R. Catmore, A. Cattai, J. Caudron, V. Cavaliere, D. Cavalli, M. Cavalli-Sforza, V. Cavasinni, F. Ceradini, B. C. Cerio, K. Cerny, A. S. Cerqueira, A. Cerri, L. Cerrito, F. Cerutti, M. Cerv, A. Cervelli, S. A. Cetin, A. Chafaq, D. Chakraborty, I. Chalupkova, P. Chang, J. D. Chapman, D. G. Charlton, C. C. Chau, C. A. Chavez Barajas, S. Cheatham, A. Chegwidden, S. Chekanov, S. V. Chekulaev, G. A. Chelkov, M. A. Chelstowska, C. Chen, H. Chen, K. Chen, L. Chen, S. Chen, X. Chen, Y. Chen, H. C. Cheng, Y. Cheng, A. Cheplakov, E. Cheremushkina, R. Cherkaoui El Moursli, V. Chernyatin, E. Cheu, L. Chevalier, V. Chiarella, G. Chiarelli, G. Chiodini, A. S. Chisholm, R. T. Chislett, A. Chitan, M. V. Chizhov, K. Choi, S. Chouridou, B. K. B. Chow, V. Christodoulou, D. Chromek-Burckhart, J. Chudoba, A. J. Chuinard, J. J. Chwastowski, L. Chytka, G. Ciapetti, A. K. Ciftci, D. Cinca, V. Cindro, I. A. Cioara, A. Ciocio, F. Cirotto, Z. H. Citron, M. Ciubancan, A. Clark, B. L. Clark, P. J. Clark, R. N. Clarke, W. Cleland, C. Clement, Y. Coadou, M. Cobal, A. Coccaro, J. Cochran, L. Coffey, J. G. Cogan, L. Colasurdo, B. Cole, S. Cole, A. P. Colijn, J. Collot, T. Colombo, G. Compostella, P. Conde Muiño, E. Coniavitis, S. H. Connell, I. A. Connelly, V. Consorti, S. Constantinescu, C. Conta, G. Conti, F. Conventi, M. Cooke, B. D. Cooper, A. M. Cooper-Sarkar, T. Cornelissen, M. Corradi, F. Corriveau, A. Corso-Radu, A. Cortes-Gonzalez, G. Cortiana, G. Costa, M. J. Costa, D. Costanzo, D. Côté, G. Cottin, G. Cowan, B. E. Cox, K. Cranmer, G. Cree, S. Crépé-Renaudin, F. Crescioli, W. A. Cribbs, M. Crispin Ortuzar, M. Cristinziani, V. Croft, G. Crosetti, T. Cuhadar Donszelmann, J. Cummings, M. Curatolo, C. Cuthbert, H. Czirr, P. Czodrowski, S. D’Auria, M. D’Onofrio, M. J. Da Cunha Sargedas De Sousa, C. Da Via, W. Dabrowski, A. Dafinca, T. Dai, O. Dale, F. Dallaire, C. Dallapiccola, M. Dam, J. R. Dandoy, N. P. Dang, A. C. Daniells, M. Danninger, M. Dano Hoffmann, V. Dao, G. Darbo, S. Darmora, J. Dassoulas, A. Dattagupta, W. Davey, C. David, T. Davidek, E. Davies, M. Davies, P. Davison, Y. Davygora, E. Dawe, I. Dawson, R. K. Daya-Ishmukhametova, K. De, R. de Asmundis, A. De Benedetti, S. De Castro, S. De Cecco, N. De Groot, P. de Jong, H. De la Torre, F. De Lorenzi, D. De Pedis, A. De Salvo, U. De Sanctis, A. De Santo, J. B. De Vivie De Regie, W. J. Dearnaley, R. Debbe, C. Debenedetti, D. V. Dedovich, I. Deigaard, J. Del Peso, T. Del Prete, D. Delgove, F. Deliot, C. M. Delitzsch, M. Deliyergiyev, A. Dell’Acqua, L. Dell’Asta, M. Dell’Orso, M. Della Pietra, D. della Volpe, M. Delmastro, P. A. Delsart, C. Deluca, D. A. DeMarco, S. Demers, M. Demichev, A. Demilly, S. P. Denisov, D. Derendarz, J. E. Derkaoui, F. Derue, P. Dervan, K. Desch, C. Deterre, P. O. Deviveiros, A. Dewhurst, S. Dhaliwal, A. Di Ciaccio, L. Di Ciaccio, A. Di Domenico, C. Di Donato, A. Di Girolamo, B. Di Girolamo, A. Di Mattia, B. Di Micco, R. Di Nardo, A. Di Simone, R. Di Sipio, D. Di Valentino, C. Diaconu, M. Diamond, F. A. Dias, M. A. Diaz, E. B. Diehl, J. Dietrich, S. Diglio, A. Dimitrievska, J. Dingfelder, P. Dita, S. Dita, F. Dittus, F. Djama, T. Djobava, J. I. Djuvsland, M. A. B. do Vale, D. Dobos, M. Dobre, C. Doglioni, T. Dohmae, J. Dolejsi, Z. Dolezal, B. A. Dolgoshein, M. Donadelli, S. Donati, P. Dondero, J. Donini, J. Dopke, A. Doria, M. T. Dova, A. T. Doyle, E. Drechsler, M. Dris, E. Dubreuil, E. Duchovni, G. Duckeck, O. A. Ducu, D. Duda, A. Dudarev, L. Duflot, L. Duguid, M. Dührssen, M. Dunford, H. Duran Yildiz, M. Düren, A. Durglishvili, D. Duschinger, M. Dyndal, C. Eckardt, K. M. Ecker, R. C. Edgar, W. Edson, N. C. Edwards, W. Ehrenfeld, T. Eifert, G. Eigen, K. Einsweiler, T. Ekelof, M. El Kacimi, M. Ellert, S. Elles, F. Ellinghaus, A. A. Elliot, N. Ellis, J. Elmsheuser, M. Elsing, D. Emeliyanov, Y. Enari, O. C. Endner, M. Endo, J. Erdmann, A. Ereditato, G. Ernis, J. Ernst, M. Ernst, S. Errede, E. Ertel, M. Escalier, H. Esch, C. Escobar, B. Esposito, A. I. Etienvre, E. Etzion, H. Evans, A. Ezhilov, L. Fabbri, G. Facini, R. M. Fakhrutdinov, S. Falciano, R. J. Falla, J. Faltova, Y. Fang, M. Fanti, A. Farbin, A. Farilla, T. Farooque, S. Farrell, S. M. Farrington, P. Farthouat, F. Fassi, P. Fassnacht, D. Fassouliotis, M. Faucci Giannelli, A. Favareto, L. Fayard, P. Federic, O. L. Fedin, W. Fedorko, S. Feigl, L. Feligioni, C. Feng, E. J. Feng, H. Feng, A. B. Fenyuk, L. Feremenga, P. Fernandez Martinez, S. Fernandez Perez, J. Ferrando, A. Ferrari, P. Ferrari, R. Ferrari, D. E. Ferreira de Lima, A. Ferrer, D. Ferrere, C. Ferretti, A. Ferretto Parodi, M. Fiascaris, F. Fiedler, A. Filipčič, M. Filipuzzi, F. Filthaut, M. Fincke-Keeler, K. D. Finelli, M. C. N. Fiolhais, L. Fiorini, A. Firan, A. Fischer, C. Fischer, J. Fischer, W. C. Fisher, E. A. Fitzgerald, N. Flaschel, I. Fleck, P. Fleischmann, S. Fleischmann, G. T. Fletcher, G. Fletcher, R. R. M. Fletcher, T. Flick, A. Floderus, L. R. Flores Castillo, M. J. Flowerdew, A. Formica, A. Forti, D. Fournier, H. Fox, S. Fracchia, P. Francavilla, M. Franchini, D. Francis, L. Franconi, M. Franklin, M. Frate, M. Fraternali, D. Freeborn, S. T. French, F. Friedrich, D. Froidevaux, J. A. Frost, C. Fukunaga, E. Fullana Torregrosa, B. G. Fulsom, T. Fusayasu, J. Fuster, C. Gabaldon, O. Gabizon, A. Gabrielli, A. Gabrielli, G. P. Gach, S. Gadatsch, S. Gadomski, G. Gagliardi, P. Gagnon, C. Galea, B. Galhardo, E. J. Gallas, B. J. Gallop, P. Gallus, G. Galster, K. K. Gan, J. Gao, Y. Gao, Y. S. Gao, F. M. Garay Walls, F. Garberson, C. García, J. E. García Navarro, M. Garcia-Sciveres, R. W. Gardner, N. Garelli, V. Garonne, C. Gatti, A. Gaudiello, G. Gaudio, B. Gaur, L. Gauthier, P. Gauzzi, I. L. Gavrilenko, C. Gay, G. Gaycken, E. N. Gazis, P. Ge, Z. Gecse, C. N. P. Gee, Ch. Geich-Gimbel, M. P. Geisler, C. Gemme, M. H. Genest, S. Gentile, M. George, S. George, D. Gerbaudo, A. Gershon, S. Ghasemi, H. Ghazlane, B. Giacobbe, S. Giagu, V. Giangiobbe, P. Giannetti, B. Gibbard, S. M. Gibson, M. Gilchriese, T. P. S. Gillam, D. Gillberg, G. Gilles, D. M. Gingrich, N. Giokaris, M. P. Giordani, F. M. Giorgi, F. M. Giorgi, P. F. Giraud, P. Giromini, D. Giugni, C. Giuliani, M. Giulini, B. K. Gjelsten, S. Gkaitatzis, I. Gkialas, E. L. Gkougkousis, L. K. Gladilin, C. Glasman, J. Glatzer, P. C. F. Glaysher, A. Glazov, M. Goblirsch-Kolb, J. R. Goddard, J. Godlewski, S. Goldfarb, T. Golling, D. Golubkov, A. Gomes, R. Gonçalo, J. Goncalves Pinto Firmino Da Costa, L. Gonella, S. González de la Hoz, G. Gonzalez Parra, S. Gonzalez-Sevilla, L. Goossens, P. A. Gorbounov, H. A. Gordon, I. Gorelov, B. Gorini, E. Gorini, A. Gorišek, E. Gornicki, A. T. Goshaw, C. Gössling, M. I. Gostkin, D. Goujdami, A. G. Goussiou, N. Govender, E. Gozani, H. M. X. Grabas, L. Graber, I. Grabowska-Bold, P. O. J. Gradin, P. Grafström, K-J. Grahn, J. Gramling, E. Gramstad, S. Grancagnolo, V. Gratchev, H. M. Gray, E. Graziani, Z. D. Greenwood, C. Grefe, K. Gregersen, I. M. Gregor, P. Grenier, J. Griffiths, A. A. Grillo, K. Grimm, S. Grinstein, Ph. Gris, J.-F. Grivaz, J. P. Grohs, A. Grohsjean, E. Gross, J. Grosse-Knetter, G. C. Grossi, Z. J. Grout, L. Guan, J. Guenther, F. Guescini, D. Guest, O. Gueta, E. Guido, T. Guillemin, S. Guindon, U. Gul, C. Gumpert, J. Guo, Y. Guo, S. Gupta, G. Gustavino, P. Gutierrez, N. G. Gutierrez Ortiz, C. Gutschow, C. Guyot, C. Gwenlan, C. B. Gwilliam, A. Haas, C. Haber, H. K. Hadavand, N. Haddad, P. Haefner, S. Hageböck, Z. Hajduk, H. Hakobyan, M. Haleem, J. Haley, D. Hall, G. Halladjian, G. D. Hallewell, K. Hamacher, P. Hamal, K. Hamano, A. Hamilton, G. N. Hamity, P. G. Hamnett, L. Han, K. Hanagaki, K. Hanawa, M. Hance, P. Hanke, R. Hanna, J. B. Hansen, J. D. Hansen, M. C. Hansen, P. H. Hansen, K. Hara, A. S. Hard, T. Harenberg, F. Hariri, S. Harkusha, R. D. Harrington, P. F. Harrison, F. Hartjes, M. Hasegawa, Y. Hasegawa, A. Hasib, S. Hassani, S. Haug, R. Hauser, L. Hauswald, M. Havranek, C. M. Hawkes, R. J. Hawkings, A. D. Hawkins, T. Hayashi, D. Hayden, C. P. Hays, J. M. Hays, H. S. Hayward, S. J. Haywood, S. J. Head, T. Heck, V. Hedberg, L. Heelan, S. Heim, T. Heim, B. Heinemann, L. Heinrich, J. Hejbal, L. Helary, S. Hellman, D. Hellmich, C. Helsens, J. Henderson, R. C. W. Henderson, Y. Heng, C. Hengler, S. Henkelmann, A. Henrichs, A. M. Henriques Correia, S. Henrot-Versille, G. H. Herbert, Y. Hernández Jiménez, R. Herrberg-Schubert, G. Herten, R. Hertenberger, L. Hervas, G. G. Hesketh, N. P. Hessey, J. W. Hetherly, R. Hickling, E. Higón-Rodriguez, E. Hill, J. C. Hill, K. H. Hiller, S. J. Hillier, I. Hinchliffe, E. Hines, R. R. Hinman, M. Hirose, D. Hirschbuehl, J. Hobbs, N. Hod, M. C. Hodgkinson, P. Hodgson, A. Hoecker, M. R. Hoeferkamp, F. Hoenig, M. Hohlfeld, D. Hohn, T. R. Holmes, M. Homann, T. M. Hong, L. Hooft van Huysduynen, W. H. Hopkins, Y. Horii, A. J. Horton, J-Y. Hostachy, S. Hou, A. Hoummada, J. Howard, J. Howarth, M. Hrabovsky, I. Hristova, J. Hrivnac, T. Hryn’ova, A. Hrynevich, C. Hsu, P. J. Hsu, S.-C. Hsu, D. Hu, Q. Hu, X. Hu, Y. Huang, Z. Hubacek, F. Hubaut, F. Huegging, T. B. Huffman, E. W. Hughes, G. Hughes, M. Huhtinen, T. A. Hülsing, N. Huseynov, J. Huston, J. Huth, G. Iacobucci, G. Iakovidis, I. Ibragimov, L. Iconomidou-Fayard, E. Ideal, Z. Idrissi, P. Iengo, O. Igonkina, T. Iizawa, Y. Ikegami, K. Ikematsu, M. Ikeno, Y. Ilchenko, D. Iliadis, N. Ilic, T. Ince, G. Introzzi, P. Ioannou, M. Iodice, K. Iordanidou, V. Ippolito, A. Irles Quiles, C. Isaksson, M. Ishino, M. Ishitsuka, R. Ishmukhametov, C. Issever, S. Istin, J. M. Iturbe Ponce, R. Iuppa, J. Ivarsson, W. Iwanski, H. Iwasaki, J. M. Izen, V. Izzo, S. Jabbar, B. Jackson, M. Jackson, P. Jackson, M. R. Jaekel, V. Jain, K. Jakobs, S. Jakobsen, T. Jakoubek, J. Jakubek, D. O. Jamin, D. K. Jana, E. Jansen, R. Jansky, J. Janssen, M. Janus, G. Jarlskog, N. Javadov, T. Javůrek, L. Jeanty, J. Jejelava, G.-Y. Jeng, D. Jennens, P. Jenni, J. Jentzsch, C. Jeske, S. Jézéquel, H. Ji, J. Jia, Y. Jiang, S. Jiggins, J. Jimenez Pena, S. Jin, A. Jinaru, O. Jinnouchi, M. D. Joergensen, P. Johansson, K. A. Johns, K. Jon-And, G. Jones, R. W. L. Jones, T. J. Jones, J. Jongmanns, P. M. Jorge, K. D. Joshi, J. Jovicevic, X. Ju, C. A. Jung, P. Jussel, A. Juste Rozas, M. Kaci, A. Kaczmarska, M. Kado, H. Kagan, M. Kagan, S. J. Kahn, E. Kajomovitz, C. W. Kalderon, S. Kama, A. Kamenshchikov, N. Kanaya, S. Kaneti, V. A. Kantserov, J. Kanzaki, B. Kaplan, L. S. Kaplan, A. Kapliy, D. Kar, K. Karakostas, A. Karamaoun, N. Karastathis, M. J. Kareem, E. Karentzos, M. Karnevskiy, S. N. Karpov, Z. M. Karpova, K. Karthik, V. Kartvelishvili, A. N. Karyukhin, L. Kashif, R. D. Kass, A. Kastanas, Y. Kataoka, C. Kato, A. Katre, J. Katzy, K. Kawagoe, T. Kawamoto, G. Kawamura, S. Kazama, V. F. Kazanin, R. Keeler, R. Kehoe, J. S. Keller, J. J. Kempster, H. Keoshkerian, O. Kepka, B. P. Kerševan, S. Kersten, R. A. Keyes, F. Khalil-zada, H. Khandanyan, A. Khanov, A. G. Kharlamov, T. J. Khoo, V. Khovanskiy, E. Khramov, J. Khubua, S. Kido, H. Y. Kim, S. H. Kim, Y. K. Kim, N. Kimura, O. M. Kind, B. T. King, M. King, S. B. King, J. Kirk, A. E. Kiryunin, T. Kishimoto, D. Kisielewska, F. Kiss, K. Kiuchi, O. Kivernyk, E. Kladiva, M. H. Klein, M. Klein, U. Klein, K. Kleinknecht, P. Klimek, A. Klimentov, R. Klingenberg, J. A. Klinger, T. Klioutchnikova, E.-E. Kluge, P. Kluit, S. Kluth, J. Knapik, E. Kneringer, E. B. F. G. Knoops, A. Knue, A. Kobayashi, D. Kobayashi, T. Kobayashi, M. Kobel, M. Kocian, P. Kodys, T. Koffas, E. Koffeman, L. A. Kogan, S. Kohlmann, Z. Kohout, T. Kohriki, T. Koi, H. Kolanoski, I. Koletsou, A. A. Komar, Y. Komori, T. Kondo, N. Kondrashova, K. Köneke, A. C. König, T. Kono, R. Konoplich, N. Konstantinidis, R. Kopeliansky, S. Koperny, L. Köpke, A. K. Kopp, K. Korcyl, K. Kordas, A. Korn, A. A. Korol, I. Korolkov, E. V. Korolkova, O. Kortner, S. Kortner, T. Kosek, V. V. Kostyukhin, V. M. Kotov, A. Kotwal, A. Kourkoumeli-Charalampidi, C. Kourkoumelis, V. Kouskoura, A. Koutsman, R. Kowalewski, T. Z. Kowalski, W. Kozanecki, A. S. Kozhin, V. A. Kramarenko, G. Kramberger, D. Krasnopevtsev, M. W. Krasny, A. Krasznahorkay, J. K. Kraus, A. Kravchenko, S. Kreiss, M. Kretz, J. Kretzschmar, K. Kreutzfeldt, P. Krieger, K. Krizka, K. Kroeninger, H. Kroha, J. Kroll, J. Kroseberg, J. Krstic, U. Kruchonak, H. Krüger, N. Krumnack, A. Kruse, M. C. Kruse, M. Kruskal, T. Kubota, H. Kucuk, S. Kuday, S. Kuehn, A. Kugel, F. Kuger, A. Kuhl, T. Kuhl, V. Kukhtin, R. Kukla, Y. Kulchitsky, S. Kuleshov, M. Kuna, T. Kunigo, A. Kupco, H. Kurashige, Y. A. Kurochkin, V. Kus, E. S. Kuwertz, M. Kuze, J. Kvita, T. Kwan, D. Kyriazopoulos, A. La Rosa, J. L. La Rosa Navarro, L. La Rotonda, C. Lacasta, F. Lacava, J. Lacey, H. Lacker, D. Lacour, V. R. Lacuesta, E. Ladygin, R. Lafaye, B. Laforge, T. Lagouri, S. Lai, L. Lambourne, S. Lammers, C. L. Lampen, W. Lampl, E. Lançon, U. Landgraf, M. P. J. Landon, V. S. Lang, J. C. Lange, A. J. Lankford, F. Lanni, K. Lantzsch, A. Lanza, S. Laplace, C. Lapoire, J. F. Laporte, T. Lari, F. Lasagni Manghi, M. Lassnig, P. Laurelli, W. Lavrijsen, A. T. Law, P. Laycock, T. Lazovich, O. Le Dortz, E. Le Guirriec, E. Le Menedeu, M. LeBlanc, T. LeCompte, F. Ledroit-Guillon, C. A. Lee, S. C. Lee, L. Lee, G. Lefebvre, M. Lefebvre, F. Legger, C. Leggett, A. Lehan, G. Lehmann Miotto, X. Lei, W. A. Leight, A. Leisos, A. G. Leister, M. A. L. Leite, R. Leitner, D. Lellouch, B. Lemmer, K. J. C. Leney, T. Lenz, B. Lenzi, R. Leone, S. Leone, C. Leonidopoulos, S. Leontsinis, C. Leroy, C. G. Lester, M. Levchenko, J. Levêque, D. Levin, L. J. Levinson, M. Levy, A. Lewis, A. M. Leyko, M. Leyton, B. Li, H. Li, H. L. Li, L. Li, L. Li, S. Li, X. Li, Y. Li, Z. Liang, H. Liao, B. Liberti, A. Liblong, P. Lichard, K. Lie, J. Liebal, W. Liebig, C. Limbach, A. Limosani, S. C. Lin, T. H. Lin, F. Linde, B. E. Lindquist, J. T. Linnemann, E. Lipeles, A. Lipniacka, M. Lisovyi, T. M. Liss, D. Lissauer, A. Lister, A. M. Litke, B. Liu, D. Liu, H. Liu, J. Liu, J. B. Liu, K. Liu, L. Liu, M. Liu, M. Liu, Y. Liu, M. Livan, A. Lleres, J. Llorente Merino, S. L. Lloyd, F. Lo Sterzo, E. Lobodzinska, P. Loch, W. S. Lockman, F. K. Loebinger, A. E. Loevschall-Jensen, A. Loginov, T. Lohse, K. Lohwasser, M. Lokajicek, B. A. Long, J. D. Long, R. E. Long, K. A. Looper, L. Lopes, D. Lopez Mateos, B. Lopez Paredes, I. Lopez Paz, J. Lorenz, N. Lorenzo Martinez, M. Losada, P. J. Lösel, X. Lou, A. Lounis, J. Love, P. A. Love, N. Lu, H. J. Lubatti, C. Luci, A. Lucotte, F. Luehring, W. Lukas, L. Luminari, O. Lundberg, B. Lund-Jensen, D. Lynn, R. Lysak, E. Lytken, H. Ma, L. L. Ma, G. Maccarrone, A. Macchiolo, C. M. Macdonald, B. Maček, J. Machado Miguens, D. Macina, D. Madaffari, R. Madar, H. J. Maddocks, W. F. Mader, A. Madsen, J. Maeda, S. Maeland, T. Maeno, A. Maevskiy, E. Magradze, K. Mahboubi, J. Mahlstedt, C. Maiani, C. Maidantchik, A. A. Maier, T. Maier, A. Maio, S. Majewski, Y. Makida, N. Makovec, B. Malaescu, Pa. Malecki, V. P. Maleev, F. Malek, U. Mallik, D. Malon, C. Malone, S. Maltezos, V. M. Malyshev, S. Malyukov, J. Mamuzic, G. Mancini, B. Mandelli, L. Mandelli, I. Mandić, R. Mandrysch, J. Maneira, A. Manfredini, L. Manhaes de Andrade Filho, J. Manjarres Ramos, A. Mann, A. Manousakis-Katsikakis, B. Mansoulie, R. Mantifel, M. Mantoani, L. Mapelli, L. March, G. Marchiori, M. Marcisovsky, C. P. Marino, M. Marjanovic, D. E. Marley, F. Marroquim, S. P. Marsden, Z. Marshall, L. F. Marti, S. Marti-Garcia, B. Martin, T. A. Martin, V. J. Martin, B. Martin dit Latour, M. Martinez, S. Martin-Haugh, V. S. Martoiu, A. C. Martyniuk, M. Marx, F. Marzano, A. Marzin, L. Masetti, T. Mashimo, R. Mashinistov, J. Masik, A. L. Maslennikov, I. Massa, L. Massa, N. Massol, P. Mastrandrea, A. Mastroberardino, T. Masubuchi, P. Mättig, J. Mattmann, J. Maurer, S. J. Maxfield, D. A. Maximov, R. Mazini, S. M. Mazza, L. Mazzaferro, G. Mc Goldrick, S. P. Mc Kee, A. McCarn, R. L. McCarthy, T. G. McCarthy, N. A. McCubbin, K. W. McFarlane, J. A. Mcfayden, G. Mchedlidze, S. J. McMahon, R. A. McPherson, M. Medinnis, S. Meehan, S. Mehlhase, A. Mehta, K. Meier, C. Meineck, B. Meirose, B. R. Mellado Garcia, F. Meloni, A. Mengarelli, S. Menke, E. Meoni, K. M. Mercurio, S. Mergelmeyer, P. Mermod, L. Merola, C. Meroni, F. S. Merritt, A. Messina, J. Metcalfe, A. S. Mete, C. Meyer, C. Meyer, J-P. Meyer, J. Meyer, H. Meyer Zu Theenhausen, R. P. Middleton, S. Miglioranzi, L. Mijović, G. Mikenberg, M. Mikestikova, M. Mikuž, M. Milesi, A. Milic, D. W. Miller, C. Mills, A. Milov, D. A. Milstead, A. A. Minaenko, Y. Minami, I. A. Minashvili, A. I. Mincer, B. Mindur, M. Mineev, Y. Ming, L. M. Mir, T. Mitani, J. Mitrevski, V. A. Mitsou, A. Miucci, P. S. Miyagawa, J. U. Mjörnmark, T. Moa, K. Mochizuki, S. Mohapatra, W. Mohr, S. Molander, R. Moles-Valls, R. Monden, K. Mönig, C. Monini, J. Monk, E. Monnier, J. Montejo Berlingen, F. Monticelli, S. Monzani, R. W. Moore, N. Morange, D. Moreno, M. Moreno Llácer, P. Morettini, D. Mori, M. Morii, M. Morinaga, V. Morisbak, S. Moritz, A. K. Morley, G. Mornacchi, J. D. Morris, S. S. Mortensen, A. Morton, L. Morvaj, M. Mosidze, J. Moss, K. Motohashi, R. Mount, E. Mountricha, S. V. Mouraviev, E. J. W. Moyse, S. Muanza, R. D. Mudd, F. Mueller, J. Mueller, R. S. P. Mueller, T. Mueller, D. Muenstermann, P. Mullen, G. A. Mullier, J. A. Murillo Quijada, W. J. Murray, H. Musheghyan, E. Musto, A. G. Myagkov, M. Myska, B. P. Nachman, O. Nackenhorst, J. Nadal, K. Nagai, R. Nagai, Y. Nagai, K. Nagano, A. Nagarkar, Y. Nagasaka, K. Nagata, M. Nagel, E. Nagy, A. M. Nairz, Y. Nakahama, K. Nakamura, T. Nakamura, I. Nakano, H. Namasivayam, R. F. Naranjo Garcia, R. Narayan, D. I. Narrias Villar, T. Naumann, G. Navarro, R. Nayyar, H. A. Neal, P. Yu. Nechaeva, T. J. Neep, P. D. Nef, A. Negri, M. Negrini, S. Nektarijevic, C. Nellist, A. Nelson, S. Nemecek, P. Nemethy, A. A. Nepomuceno, M. Nessi, M. S. Neubauer, M. Neumann, R. M. Neves, P. Nevski, P. R. Newman, D. H. Nguyen, R. B. Nickerson, R. Nicolaidou, B. Nicquevert, J. Nielsen, N. Nikiforou, A. Nikiforov, V. Nikolaenko, I. Nikolic-Audit, K. Nikolopoulos, J. K. Nilsen, P. Nilsson, Y. Ninomiya, A. Nisati, R. Nisius, T. Nobe, M. Nomachi, I. Nomidis, T. Nooney, S. Norberg, M. Nordberg, O. Novgorodova, S. Nowak, M. Nozaki, L. Nozka, K. Ntekas, G. Nunes Hanninger, T. Nunnemann, E. Nurse, F. Nuti, B. J. O’Brien, F. O’grady, D. C. O’Neil, V. O’Shea, F. G. Oakham, H. Oberlack, T. Obermann, J. Ocariz, A. Ochi, I. Ochoa, J. P. Ochoa-Ricoux, S. Oda, S. Odaka, H. Ogren, A. Oh, S. H. Oh, C. C. Ohm, H. Ohman, H. Oide, W. Okamura, H. Okawa, Y. Okumura, T. Okuyama, A. Olariu, S. A. Olivares Pino, D. Oliveira Damazio, E. Oliver Garcia, A. Olszewski, J. Olszowska, A. Onofre, K. Onogi, P. U. E. Onyisi, C. J. Oram, M. J. Oreglia, Y. Oren, D. Orestano, N. Orlando, C. Oropeza Barrera, R. S. Orr, B. Osculati, R. Ospanov, G. Otero y Garzon, H. Otono, M. Ouchrif, F. Ould-Saada, A. Ouraou, K. P. Oussoren, Q. Ouyang, A. Ovcharova, M. Owen, R. E. Owen, V. E. Ozcan, N. Ozturk, K. Pachal, A. Pacheco Pages, C. Padilla Aranda, M. Pagáčová, S. Pagan Griso, E. Paganis, F. Paige, P. Pais, K. Pajchel, G. Palacino, S. Palestini, M. Palka, D. Pallin, A. Palma, Y. B. Pan, E. Panagiotopoulou, C. E. Pandini, J. G. Panduro Vazquez, P. Pani, S. Panitkin, D. Pantea, L. Paolozzi, Th. D. Papadopoulou, K. Papageorgiou, A. Paramonov, D. Paredes Hernandez, M. A. Parker, K. A. Parker, F. Parodi, J. A. Parsons, U. Parzefall, E. Pasqualucci, S. Passaggio, F. Pastore, Fr. Pastore, G. Pásztor, S. Pataraia, N. D. Patel, J. R. Pater, T. Pauly, J. Pearce, B. Pearson, L. E. Pedersen, M. Pedersen, S. Pedraza Lopez, R. Pedro, S. V. Peleganchuk, D. Pelikan, O. Penc, C. Peng, H. Peng, B. Penning, J. Penwell, D. V. Perepelitsa, E. Perez Codina, M. T. Pérez García-Estañ, L. Perini, H. Pernegger, S. Perrella, R. Peschke, V. D. Peshekhonov, K. Peters, R. F. Y. Peters, B. A. Petersen, T. C. Petersen, E. Petit, A. Petridis, C. Petridou, P. Petroff, E. Petrolo, F. Petrucci, N. E. Pettersson, R. Pezoa, P. W. Phillips, G. Piacquadio, E. Pianori, A. Picazio, E. Piccaro, M. Piccinini, M. A. Pickering, R. Piegaia, D. T. Pignotti, J. E. Pilcher, A. D. Pilkington, J. Pina, M. Pinamonti, J. L. Pinfold, A. Pingel, S. Pires, H. Pirumov, M. Pitt, C. Pizio, L. Plazak, M.-A. Pleier, V. Pleskot, E. Plotnikova, P. Plucinski, D. Pluth, R. Poettgen, L. Poggioli, D. Pohl, G. Polesello, A. Poley, A. Policicchio, R. Polifka, A. Polini, C. S. Pollard, V. Polychronakos, K. Pommès, L. Pontecorvo, B. G. Pope, G. A. Popeneciu, D. S. Popovic, A. Poppleton, S. Pospisil, K. Potamianos, I. N. Potrap, C. J. Potter, C. T. Potter, G. Poulard, J. Poveda, V. Pozdnyakov, P. Pralavorio, A. Pranko, S. Prasad, S. Prell, D. Price, L. E. Price, M. Primavera, S. Prince, M. Proissl, K. Prokofiev, F. Prokoshin, E. Protopapadaki, S. Protopopescu, J. Proudfoot, M. Przybycien, E. Ptacek, D. Puddu, E. Pueschel, D. Puldon, M. Purohit, P. Puzo, J. Qian, G. Qin, Y. Qin, A. Quadt, D. R. Quarrie, W. B. Quayle, M. Queitsch-Maitland, D. Quilty, S. Raddum, V. Radeka, V. Radescu, S. K. Radhakrishnan, P. Radloff, P. Rados, F. Ragusa, G. Rahal, S. Rajagopalan, M. Rammensee, C. Rangel-Smith, F. Rauscher, S. Rave, T. Ravenscroft, M. Raymond, A. L. Read, N. P. Readioff, D. M. Rebuzzi, A. Redelbach, G. Redlinger, R. Reece, K. Reeves, L. Rehnisch, J. Reichert, H. Reisin, M. Relich, C. Rembser, H. Ren, A. Renaud, M. Rescigno, S. Resconi, O. L. Rezanova, P. Reznicek, R. Rezvani, R. Richter, S. Richter, E. Richter-Was, O. Ricken, M. Ridel, P. Rieck, C. J. Riegel, J. Rieger, O. Rifki, M. Rijssenbeek, A. Rimoldi, L. Rinaldi, B. Ristić, E. Ritsch, I. Riu, F. Rizatdinova, E. Rizvi, S. H. Robertson, A. Robichaud-Veronneau, D. Robinson, J. E. M. Robinson, A. Robson, C. Roda, S. Roe, O. Røhne, S. Rolli, A. Romaniouk, M. Romano, S. M. Romano Saez, E. Romero Adam, N. Rompotis, M. Ronzani, L. Roos, E. Ros, S. Rosati, K. Rosbach, P. Rose, P. L. Rosendahl, O. Rosenthal, V. Rossetti, E. Rossi, L. P. Rossi, J. H. N. Rosten, R. Rosten, M. Rotaru, I. Roth, J. Rothberg, D. Rousseau, C. R. Royon, A. Rozanov, Y. Rozen, X. Ruan, F. Rubbo, I. Rubinskiy, V. I. Rud, C. Rudolph, M. S. Rudolph, F. Rühr, A. Ruiz-Martinez, Z. Rurikova, N. A. Rusakovich, A. Ruschke, H. L. Russell, J. P. Rutherfoord, N. Ruthmann, Y. F. Ryabov, M. Rybar, G. Rybkin, N. C. Ryder, A. F. Saavedra, G. Sabato, S. Sacerdoti, A. Saddique, H. F-W. Sadrozinski, R. Sadykov, F. Safai Tehrani, M. Sahinsoy, M. Saimpert, T. Saito, H. Sakamoto, Y. Sakurai, G. Salamanna, A. Salamon, J. E. Salazar Loyola, M. Saleem, D. Salek, P. H. Sales De Bruin, D. Salihagic, A. Salnikov, J. Salt, D. Salvatore, F. Salvatore, A. Salvucci, A. Salzburger, D. Sammel, D. Sampsonidis, A. Sanchez, J. Sánchez, V. Sanchez Martinez, H. Sandaker, R. L. Sandbach, H. G. Sander, M. P. Sanders, M. Sandhoff, C. Sandoval, R. Sandstroem, D. P. C. Sankey, M. Sannino, A. Sansoni, C. Santoni, R. Santonico, H. Santos, I. Santoyo Castillo, K. Sapp, A. Sapronov, J. G. Saraiva, B. Sarrazin, O. Sasaki, Y. Sasaki, K. Sato, G. Sauvage, E. Sauvan, G. Savage, P. Savard, C. Sawyer, L. Sawyer, J. Saxon, C. Sbarra, A. Sbrizzi, T. Scanlon, D. A. Scannicchio, M. Scarcella, V. Scarfone, J. Schaarschmidt, P. Schacht, D. Schaefer, R. Schaefer, J. Schaeffer, S. Schaepe, S. Schaetzel, U. Schäfer, A. C. Schaffer, D. Schaile, R. D. Schamberger, V. Scharf, V. A. Schegelsky, D. Scheirich, M. Schernau, C. Schiavi, C. Schillo, M. Schioppa, S. Schlenker, K. Schmieden, C. Schmitt, S. Schmitt, S. Schmitt, B. Schneider, Y. J. Schnellbach, U. Schnoor, L. Schoeffel, A. Schoening, B. D. Schoenrock, E. Schopf, A. L. S. Schorlemmer, M. Schott, D. Schouten, J. Schovancova, S. Schramm, M. Schreyer, C. Schroeder, N. Schuh, M. J. Schultens, H.-C. Schultz-Coulon, H. Schulz, M. Schumacher, B. A. Schumm, Ph. Schune, C. Schwanenberger, A. Schwartzman, T. A. Schwarz, Ph. Schwegler, H. Schweiger, Ph. Schwemling, R. Schwienhorst, J. Schwindling, T. Schwindt, F. G. Sciacca, E. Scifo, G. Sciolla, F. Scuri, F. Scutti, J. Searcy, G. Sedov, E. Sedykh, P. Seema, S. C. Seidel, A. Seiden, F. Seifert, J. M. Seixas, G. Sekhniaidze, K. Sekhon, S. J. Sekula, D. M. Seliverstov, N. Semprini-Cesari, C. Serfon, L. Serin, L. Serkin, T. Serre, M. Sessa, R. Seuster, H. Severini, T. Sfiligoj, F. Sforza, A. Sfyrla, E. Shabalina, M. Shamim, L. Y. Shan, R. Shang, J. T. Shank, M. Shapiro, P. B. Shatalov, K. Shaw, S. M. Shaw, A. Shcherbakova, C. Y. Shehu, P. Sherwood, L. Shi, S. Shimizu, C. O. Shimmin, M. Shimojima, M. Shiyakova, A. Shmeleva, D. Shoaleh Saadi, M. J. Shochet, S. Shojaii, S. Shrestha, E. Shulga, M. A. Shupe, S. Shushkevich, P. Sicho, P. E. Sidebo, O. Sidiropoulou, D. Sidorov, A. Sidoti, F. Siegert, Dj. Sijacki, J. Silva, Y. Silver, S. B. Silverstein, V. Simak, O. Simard, Lj. Simic, S. Simion, E. Simioni, B. Simmons, D. Simon, P. Sinervo, N. B. Sinev, M. Sioli, G. Siragusa, A. N. Sisakyan, S. Yu. Sivoklokov, J. Sjölin, T. B. Sjursen, M. B. Skinner, H. P. Skottowe, P. Skubic, M. Slater, T. Slavicek, M. Slawinska, K. Sliwa, V. Smakhtin, B. H. Smart, L. Smestad, S. Yu. Smirnov, Y. Smirnov, L. N. Smirnova, O. Smirnova, M. N. K. Smith, R. W. Smith, M. Smizanska, K. Smolek, A. A. Snesarev, G. Snidero, S. Snyder, R. Sobie, F. Socher, A. Soffer, D. A. Soh, G. Sokhrannyi, C. A. Solans, M. Solar, J. Solc, E. Yu. Soldatov, U. Soldevila, A. A. Solodkov, A. Soloshenko, O. V. Solovyanov, V. Solovyev, P. Sommer, H. Y. Song, N. Soni, A. Sood, A. Sopczak, B. Sopko, V. Sopko, V. Sorin, D. Sosa, M. Sosebee, C. L. Sotiropoulou, R. Soualah, A. M. Soukharev, D. South, B. C. Sowden, S. Spagnolo, M. Spalla, M. Spangenberg, F. Spanò, W. R. Spearman, D. Sperlich, F. Spettel, R. Spighi, G. Spigo, L. A. Spiller, M. Spousta, T. Spreitzer, R. D. St. Denis, A. Stabile, S. Staerz, J. Stahlman, R. Stamen, S. Stamm, E. Stanecka, C. Stanescu, M. Stanescu-Bellu, M. M. Stanitzki, S. Stapnes, E. A. Starchenko, J. Stark, P. Staroba, P. Starovoitov, R. Staszewski, P. Steinberg, B. Stelzer, H. J. Stelzer, O. Stelzer-Chilton, H. Stenzel, G. A. Stewart, J. A. Stillings, M. C. Stockton, M. Stoebe, G. Stoicea, P. Stolte, S. Stonjek, A. R. Stradling, A. Straessner, M. E. Stramaglia, J. Strandberg, S. Strandberg, A. Strandlie, E. Strauss, M. Strauss, P. Strizenec, R. Ströhmer, D. M. Strom, R. Stroynowski, A. Strubig, S. A. Stucci, B. Stugu, N. A. Styles, D. Su, J. Su, R. Subramaniam, A. Succurro, Y. Sugaya, M. Suk, V. V. Sulin, S. Sultansoy, T. Sumida, S. Sun, X. Sun, J. E. Sundermann, K. Suruliz, G. Susinno, M. R. Sutton, S. Suzuki, M. Svatos, M. Swiatlowski, I. Sykora, T. Sykora, D. Ta, C. Taccini, K. Tackmann, J. Taenzer, A. Taffard, R. Tafirout, N. Taiblum, H. Takai, R. Takashima, H. Takeda, T. Takeshita, Y. Takubo, M. Talby, A. A. Talyshev, J. Y. C. Tam, K. G. Tan, J. Tanaka, R. Tanaka, S. Tanaka, B. B. Tannenwald, N. Tannoury, S. Tapprogge, S. Tarem, F. Tarrade, G. F. Tartarelli, P. Tas, M. Tasevsky, T. Tashiro, E. Tassi, A. Tavares Delgado, Y. Tayalati, F. E. Taylor, G. N. Taylor, W. Taylor, F. A. Teischinger, M. Teixeira Dias Castanheira, P. Teixeira-Dias, K. K. Temming, D. Temple, H. Ten Kate, P. K. Teng, J. J. Teoh, F. Tepel, S. Terada, K. Terashi, J. Terron, S. Terzo, M. Testa, R. J. Teuscher, T. Theveneaux-Pelzer, J. P. Thomas, J. Thomas-Wilsker, E. N. Thompson, P. D. Thompson, R. J. Thompson, A. S. Thompson, L. A. Thomsen, E. Thomson, M. Thomson, R. P. Thun, M. J. Tibbetts, R. E. Ticse Torres, V. O. Tikhomirov, Yu. A. Tikhonov, S. Timoshenko, E. Tiouchichine, P. Tipton, S. Tisserant, K. Todome, T. Todorov, S. Todorova-Nova, J. Tojo, S. Tokár, K. Tokushuku, K. Tollefson, E. Tolley, L. Tomlinson, M. Tomoto, L. Tompkins, K. Toms, E. Torrence, H. Torres, E. Torró Pastor, J. Toth, F. Touchard, D. R. Tovey, T. Trefzger, L. Tremblet, A. Tricoli, I. M. Trigger, S. Trincaz-Duvoid, M. F. Tripiana, W. Trischuk, B. Trocmé, C. Troncon, M. Trottier-McDonald, M. Trovatelli, P. True, L. Truong, M. Trzebinski, A. Trzupek, C. Tsarouchas, J. C-L. Tseng, P. V. Tsiareshka, D. Tsionou, G. Tsipolitis, N. Tsirintanis, S. Tsiskaridze, V. Tsiskaridze, E. G. Tskhadadze, I. I. Tsukerman, V. Tsulaia, S. Tsuno, D. Tsybychev, A. Tudorache, V. Tudorache, A. N. Tuna, S. A. Tupputi, S. Turchikhin, D. Turecek, R. Turra, A. J. Turvey, P. M. Tuts, A. Tykhonov, M. Tylmad, M. Tyndel, I. Ueda, R. Ueno, M. Ughetto, M. Ugland, F. Ukegawa, G. Unal, A. Undrus, G. Unel, F. C. Ungaro, Y. Unno, C. Unverdorben, J. Urban, P. Urquijo, P. Urrejola, G. Usai, A. Usanova, L. Vacavant, V. Vacek, B. Vachon, C. Valderanis, N. Valencic, S. Valentinetti, A. Valero, L. Valery, S. Valkar, E. Valladolid Gallego, S. Vallecorsa, J. A. Valls Ferrer, W. Van Den Wollenberg, P. C. Van Der Deijl, R. van der Geer, H. van der Graaf, N. van Eldik, P. van Gemmeren, J. Van Nieuwkoop, I. van Vulpen, M. C. van Woerden, M. Vanadia, W. Vandelli, R. Vanguri, A. Vaniachine, F. Vannucci, G. Vardanyan, R. Vari, E. W. Varnes, T. Varol, D. Varouchas, A. Vartapetian, K. E. Varvell, F. Vazeille, T. Vazquez Schroeder, J. Veatch, L. M. Veloce, F. Veloso, T. Velz, S. Veneziano, A. Ventura, D. Ventura, M. Venturi, N. Venturi, A. Venturini, V. Vercesi, M. Verducci, W. Verkerke, J. C. Vermeulen, A. Vest, M. C. Vetterli, O. Viazlo, I. Vichou, T. Vickey, O. E. Vickey Boeriu, G. H. A. Viehhauser, S. Viel, R. Vigne, M. Villa, M. Villaplana Perez, E. Vilucchi, M. G. Vincter, V. B. Vinogradov, I. Vivarelli, F. Vives Vaque, S. Vlachos, D. Vladoiu, M. Vlasak, M. Vogel, P. Vokac, G. Volpi, M. Volpi, H. von der Schmitt, H. von Radziewski, E. von Toerne, V. Vorobel, K. Vorobev, M. Vos, R. Voss, J. H. Vossebeld, N. Vranjes, M. Vranjes Milosavljevic, V. Vrba, M. Vreeswijk, R. Vuillermet, I. Vukotic, Z. Vykydal, P. Wagner, W. Wagner, H. Wahlberg, S. Wahrmund, J. Wakabayashi, J. Walder, R. Walker, W. Walkowiak, C. Wang, F. Wang, H. Wang, H. Wang, J. Wang, J. Wang, K. Wang, R. Wang, S. M. Wang, T. Wang, T. Wang, X. Wang, C. Wanotayaroj, A. Warburton, C. P. Ward, D. R. Wardrope, A. Washbrook, C. Wasicki, P. M. Watkins, A. T. Watson, I. J. Watson, M. F. Watson, G. Watts, S. Watts, B. M. Waugh, S. Webb, M. S. Weber, S. W. Weber, J. S. Webster, A. R. Weidberg, B. Weinert, J. Weingarten, C. Weiser, H. Weits, P. S. Wells, T. Wenaus, T. Wengler, S. Wenig, N. Wermes, M. Werner, P. Werner, M. Wessels, J. Wetter, K. Whalen, A. M. Wharton, A. White, M. J. White, R. White, S. White, D. Whiteson, F. J. Wickens, W. Wiedenmann, M. Wielers, P. Wienemann, C. Wiglesworth, L. A. M. Wiik-Fuchs, A. Wildauer, H. G. Wilkens, H. H. Williams, S. Williams, C. Willis, S. Willocq, A. Wilson, J. A. Wilson, I. Wingerter-Seez, F. Winklmeier, B. T. Winter, M. Wittgen, J. Wittkowski, S. J. Wollstadt, M. W. Wolter, H. Wolters, B. K. Wosiek, J. Wotschack, M. J. Woudstra, K. W. Wozniak, M. Wu, M. Wu, S. L. Wu, X. Wu, Y. Wu, T. R. Wyatt, B. M. Wynne, S. Xella, D. Xu, L. Xu, B. Yabsley, S. Yacoob, R. Yakabe, M. Yamada, D. Yamaguchi, Y. Yamaguchi, A. Yamamoto, S. Yamamoto, T. Yamanaka, K. Yamauchi, Y. Yamazaki, Z. Yan, H. Yang, H. Yang, Y. Yang, W-M. Yao, Y. Yasu, E. Yatsenko, K. H. Yau Wong, J. Ye, S. Ye, I. Yeletskikh, A. L. Yen, E. Yildirim, K. Yorita, R. Yoshida, K. Yoshihara, C. Young, C. J. S. Young, S. Youssef, D. R. Yu, J. Yu, J. M. Yu, J. Yu, L. Yuan, S. P. Y. Yuen, A. Yurkewicz, I. Yusuff, B. Zabinski, R. Zaidan, A. M. Zaitsev, J. Zalieckas, A. Zaman, S. Zambito, L. Zanello, D. Zanzi, C. Zeitnitz, M. Zeman, A. Zemla, Q. Zeng, K. Zengel, O. Zenin, T. Ženiš, D. Zerwas, D. Zhang, F. Zhang, H. Zhang, J. Zhang, L. Zhang, R. Zhang, X. Zhang, Z. Zhang, X. Zhao, Y. Zhao, Z. Zhao, A. Zhemchugov, J. Zhong, B. Zhou, C. Zhou, L. Zhou, L. Zhou, M. Zhou, N. Zhou, C. G. Zhu, H. Zhu, J. Zhu, Y. Zhu, X. Zhuang, K. Zhukov, A. Zibell, D. Zieminska, N. I. Zimine, C. Zimmermann, S. Zimmermann, Z. Zinonos, M. Zinser, M. Ziolkowski, L. Živković, G. Zobernig, A. Zoccoli, M. zur Nedden, G. Zurzolo, L. Zwalinski, ATLAS Collaboration

**Affiliations:** 1 Department of Physics, University of Adelaide, Adelaide, Australia; 2 Physics Department, SUNY Albany, Albany, NY USA; 3 Department of Physics, University of Alberta, Edmonton, AB Canada; 4 Department of Physics, Ankara University, Ankara, Turkey; 5 Istanbul Aydin University, Istanbul, Turkey; 6 Division of Physics, TOBB University of Economics and Technology, Ankara, Turkey; 7 LAPP, CNRS/IN2P3 and Université Savoie Mont Blanc, Annecy-le-Vieux, France; 8 High Energy Physics Division, Argonne National Laboratory, Argonne, IL USA; 9 Department of Physics, University of Arizona, Tucson, AZ USA; 10 Department of Physics, The University of Texas at Arlington, Arlington, TX USA; 11 Physics Department, University of Athens, Athens, Greece; 12 Physics Department, National Technical University of Athens, Zografou, Greece; 13 Institute of Physics, Azerbaijan Academy of Sciences, Baku, Azerbaijan; 14 Institut de Física d’Altes Energies and Departament de Física de la Universitat Autònoma de Barcelona, Barcelona, Spain; 15 Institute of Physics, University of Belgrade, Belgrade, Serbia; 16 Department for Physics and Technology, University of Bergen, Bergen, Norway; 17 Physics Division, Lawrence Berkeley National Laboratory and University of California, Berkeley, CA USA; 18 Department of Physics, Humboldt University, Berlin, Germany; 19 Albert Einstein Center for Fundamental Physics and Laboratory for High Energy Physics, University of Bern, Bern, Switzerland; 20 School of Physics and Astronomy, University of Birmingham, Birmingham, UK; 21 Department of Physics, Bogazici University, Istanbul, Turkey; 22 Department of Physics Engineering, Gaziantep University, Gaziantep, Turkey; 23 Department of Physics, Dogus University, Istanbul, Turkey; 24 INFN Sezione di Bologna, Bologna, Italy; 25 Dipartimento di Fisica e Astronomia, Università di Bologna, Bologna, Italy; 26 Physikalisches Institut, University of Bonn, Bonn, Germany; 27 Department of Physics, Boston University, Boston, MA USA; 28 Department of Physics, Brandeis University, Waltham, MA USA; 29 Universidade Federal do Rio De Janeiro COPPE/EE/IF, Rio de Janeiro, Brazil; 30 Electrical Circuits Department, Federal University of Juiz de Fora (UFJF), Juiz de Fora, Brazil; 31 Federal University of Sao Joao del Rei (UFSJ), Sao Joao del Rei, Brazil; 32 Instituto de Fisica, Universidade de Sao Paulo, Sao Paulo, Brazil; 33 Physics Department, Brookhaven National Laboratory, Upton, NY USA; 34 National Institute of Physics and Nuclear Engineering, Bucharest, Romania; 35 Physics Department, National Institute for Research and Development of Isotopic and Molecular Technologies, Cluj Napoca, Romania; 38 Departamento de Física, Universidad de Buenos Aires, Buenos Aires, Argentina; 39 Cavendish Laboratory, University of Cambridge, Cambridge, UK; 40 Department of Physics, Carleton University, Ottawa, ON Canada; 41 CERN, Geneva, Switzerland; 42 Enrico Fermi Institute, University of Chicago, Chicago, IL USA; 43 Departamento de Física, Pontificia Universidad Católica de Chile, Santiago, Chile; 44 Departamento de Física, Universidad Técnica Federico Santa María, Valparaiso, Chile; 45 Institute of High Energy Physics, Chinese Academy of Sciences, Beijing, China; 46 Department of Modern Physics, University of Science and Technology of China, Anhui, China; 47 Department of Physics, Nanjing University, Jiangsu, China; 48 School of Physics, Shandong University, Shandong, China; 49 Shanghai Key Laboratory for Particle Physics and Cosmology, Department of Physics and Astronomy, Shanghai Jiao Tong University, Shanghai, China; 50 Physics Department, Tsinghua University, Beijing, 100084 China; 51 Laboratoire de Physique Corpusculaire, Clermont Université and Université Blaise Pascal and CNRS/IN2P3, Clermont-Ferrand, France; 52 Nevis Laboratory, Columbia University, Irvington, NY USA; 53 Niels Bohr Institute, University of Copenhagen, Kobenhavn, Denmark; 54 INFN Gruppo Collegato di Cosenza, Laboratori Nazionali di Frascati, Frascati, Italy; 55 Dipartimento di Fisica, Università della Calabria, Rende, Italy; 56 AGH University of Science and Technology, Faculty of Physics and Applied Computer Science, Kraków, Poland; 57 Marian Smoluchowski Institute of Physics, Jagiellonian University, Kraków, Poland; 58 Institute of Nuclear Physics, Polish Academy of Sciences, Kraków, Poland; 59 Physics Department, Southern Methodist University, Dallas, TX USA; 60 Physics Department, University of Texas at Dallas, Richardson, TX USA; 61 DESY, Hamburg and Zeuthen, Germany; 62 Institut für Experimentelle Physik IV, Technische Universität Dortmund, Dortmund, Germany; 63 Institut für Kern- und Teilchenphysik, Technische Universität Dresden, Dresden, Germany; 64 Department of Physics, Duke University, Durham, NC USA; 65 SUPA-School of Physics and Astronomy, University of Edinburgh, Edinburgh, UK; 66 INFN Laboratori Nazionali di Frascati, Frascati, Italy; 67 Fakultät für Mathematik und Physik, Albert-Ludwigs-Universität, Freiburg, Germany; 68 Section de Physique, Université de Genève, Geneva, Switzerland; 69 INFN Sezione di Genova, Genoa, Italy; 70 Dipartimento di Fisica, Università di Genova, Genoa, Italy; 71 E. Andronikashvili Institute of Physics, Iv. Javakhishvili Tbilisi State University, Tbilisi, Georgia; 72 High Energy Physics Institute, Tbilisi State University, Tbilisi, Georgia; 73 II Physikalisches Institut, Justus-Liebig-Universität Giessen, Giessen, Germany; 74 SUPA-School of Physics and Astronomy, University of Glasgow, Glasgow, UK; 75 II Physikalisches Institut, Georg-August-Universität, Göttingen, Germany; 76 Laboratoire de Physique Subatomique et de Cosmologie, Université Grenoble-Alpes, CNRS/IN2P3, Grenoble, France; 77 Department of Physics, Hampton University, Hampton, VA USA; 78 Laboratory for Particle Physics and Cosmology, Harvard University, Cambridge, MA USA; 79 Kirchhoff-Institut für Physik, Ruprecht-Karls-Universität Heidelberg, Heidelberg, Germany; 80 Physikalisches Institut, Ruprecht-Karls-Universität Heidelberg, Heidelberg, Germany; 81 ZITI Institut für technische Informatik, Ruprecht-Karls-Universität Heidelberg, Mannheim, Germany; 82 Faculty of Applied Information Science, Hiroshima Institute of Technology, Hiroshima, Japan; 83 Department of Physics, The Chinese University of Hong Kong, Shatin, NT Hong Kong; 84 Department of Physics, The University of Hong Kong, Pokfulam, Hong Kong; 85 Department of Physics, The Hong Kong University of Science and Technology, Clear Water Bay, Kowloon, Hong Kong, China; 86 Department of Physics, Indiana University, Bloomington, IN USA; 87 Institut für Astro- und Teilchenphysik, Leopold-Franzens-Universität, Innsbruck, Austria; 88 University of Iowa, Iowa City, IA USA; 89 Department of Physics and Astronomy, Iowa State University, Ames, IA USA; 90 Joint Institute for Nuclear Research, JINR Dubna, Dubna, Russia; 91 KEK, High Energy Accelerator Research Organization, Tsukuba, Japan; 92 Graduate School of Science, Kobe University, Kobe, Japan; 93 Faculty of Science, Kyoto University, Kyoto, Japan; 94 Kyoto University of Education, Kyoto, Japan; 95 Department of Physics, Kyushu University, Fukuoka, Japan; 96 Instituto de Física La Plata, Universidad Nacional de La Plata and CONICET, La Plata, Argentina; 97 Physics Department, Lancaster University, Lancaster, UK; 98 INFN Sezione di Lecce, Lecce, Italy; 99 Dipartimento di Matematica e Fisica, Università del Salento, Lecce, Italy; 100 Oliver Lodge Laboratory, University of Liverpool, Liverpool, UK; 101 Department of Physics, Jožef Stefan Institute and University of Ljubljana, Ljubljana, Slovenia; 102 School of Physics and Astronomy, Queen Mary University of London, London, UK; 103 Department of Physics, Royal Holloway University of London, Surrey, UK; 104 Department of Physics and Astronomy, University College London, London, UK; 105 Louisiana Tech University, Ruston, LA USA; 106 Laboratoire de Physique Nucléaire et de Hautes Energies, UPMC and Université Paris-Diderot and CNRS/IN2P3, Paris, France; 107 Fysiska institutionen, Lunds universitet, Lund, Sweden; 108 Departamento de Fisica Teorica C-15, Universidad Autonoma de Madrid, Madrid, Spain; 109 Institut für Physik, Universität Mainz, Mainz, Germany; 110 School of Physics and Astronomy, University of Manchester, Manchester, UK; 111 CPPM, Aix-Marseille Université and CNRS/IN2P3, Marseille, France; 112 Department of Physics, University of Massachusetts, Amherst, MA USA; 113 Department of Physics, McGill University, Montreal, QC Canada; 114 School of Physics, University of Melbourne, Melbourne, Victoria Australia; 115 Department of Physics, The University of Michigan, Ann Arbor, MI USA; 116 Department of Physics and Astronomy, Michigan State University, East Lansing, MI USA; 117 INFN Sezione di Milano, Milan, Italy; 118 Dipartimento di Fisica, Università di Milano, Milan, Italy; 119 B.I. Stepanov Institute of Physics, National Academy of Sciences of Belarus, Minsk, Republic of Belarus; 120 National Scientific and Educational Centre for Particle and High Energy Physics, Minsk, Republic of Belarus; 121 Department of Physics, Massachusetts Institute of Technology, Cambridge, MA USA; 122 Group of Particle Physics, University of Montreal, Montreal, QC Canada; 123 P.N. Lebedev Institute of Physics, Academy of Sciences, Moscow, Russia; 124 Institute for Theoretical and Experimental Physics (ITEP), Moscow, Russia; 125 National Research Nuclear University MEPhI, Moscow, Russia; 126 D.V. Skobeltsyn Institute of Nuclear Physics, M.V. Lomonosov Moscow State University, Moscow, Russia; 127 Fakultät für Physik, Ludwig-Maximilians-Universität München, Munich, Germany; 128 Max-Planck-Institut für Physik (Werner-Heisenberg-Institut), Munich, Germany; 129 Nagasaki Institute of Applied Science, Nagasaki, Japan; 130 Graduate School of Science and Kobayashi-Maskawa Institute, Nagoya University, Nagoya, Japan; 131 INFN Sezione di Napoli, Naples, Italy; 132 Dipartimento di Fisica, Università di Napoli, Naples, Italy; 133 Department of Physics and Astronomy, University of New Mexico, Albuquerque, NM USA; 134 Institute for Mathematics, Astrophysics and Particle Physics, Radboud University Nijmegen/Nikhef, Nijmegen, The Netherlands; 135 Nikhef National Institute for Subatomic Physics and University of Amsterdam, Amsterdam, The Netherlands; 136 Department of Physics, Northern Illinois University, De Kalb, IL USA; 137 Budker Institute of Nuclear Physics, SB RAS, Novosibirsk, Russia; 138 Department of Physics, New York University, New York, NY USA; 139 Ohio State University, Columbus, OH USA; 140 Faculty of Science, Okayama University, Okayama, Japan; 141 Homer L. Dodge Department of Physics and Astronomy, University of Oklahoma, Norman, OK USA; 142 Department of Physics, Oklahoma State University, Stillwater, OK USA; 143 Palacký University, RCPTM, Olomouc, Czech Republic; 144 Center for High Energy Physics, University of Oregon, Eugene, OR USA; 145 LAL, Université Paris-Sud and CNRS/IN2P3, Orsay, France; 146 Graduate School of Science, Osaka University, Osaka, Japan; 147 Department of Physics, University of Oslo, Oslo, Norway; 148 Department of Physics, Oxford University, Oxford, UK; 149 INFN Sezione di Pavia, Pavia, Italy; 150 Dipartimento di Fisica, Università di Pavia, Pavia, Italy; 151 Department of Physics, University of Pennsylvania, Philadelphia, PA USA; 152 National Research Centre ”Kurchatov Institute” B.P.Konstantinov Petersburg Nuclear Physics Institute, St. Petersburg, Russia; 153 INFN Sezione di Pisa, Pisa, Italy; 154 Dipartimento di Fisica E. Fermi, Università di Pisa, Pisa, Italy; 155 Department of Physics and Astronomy, University of Pittsburgh, Pittsburgh, PA USA; 156 Laboratório de Instrumentação e Física Experimental de Partículas-LIP, Lisbon, Portugal; 157 Faculdade de Ciências, Universidade de Lisboa, Lisbon, Portugal; 158 Department of Physics, University of Coimbra, Coimbra, Portugal; 159 Centro de Física Nuclear da Universidade de Lisboa, Lisbon, Portugal; 160 Departamento de Fisica, Universidade do Minho, Braga, Portugal; 161 Departamento de Fisica Teorica y del Cosmos and CAFPE, Universidad de Granada, Granada, Spain; 163 Institute of Physics, Academy of Sciences of the Czech Republic, Praha, Czech Republic; 164 Czech Technical University in Prague, Praha, Czech Republic; 165 Faculty of Mathematics and Physics, Charles University in Prague, Prague, Czech Republic; 166 State Research Center Institute for High Energy Physics, Protvino, Russia; 167 Particle Physics Department, Rutherford Appleton Laboratory, Didcot, UK; 168 INFN Sezione di Roma, Rome, Italy; 169 Dipartimento di Fisica, Sapienza Università di Roma, Rome, Italy; 170 INFN Sezione di Roma Tor Vergata, Rome, Italy; 171 Dipartimento di Fisica, Università di Roma Tor Vergata, Rome, Italy; 172 INFN Sezione di Roma Tre, Rome, Italy; 173 Dipartimento di Matematica e Fisica, Università Roma Tre, Rome, Italy; 174 Faculté des Sciences Ain Chock, Réseau Universitaire de Physique des Hautes Energies-Université Hassan II, Casablanca, Morocco; 175 Centre National de l’Energie des Sciences Techniques Nucleaires, Rabat, Morocco; 176 Faculté des Sciences Semlalia, Université Cadi Ayyad, LPHEA-Marrakech, Marrakech, Morocco; 177 Faculté des Sciences, Université Mohamed Premier and LPTPM, Oujda, Morocco; 178 Faculté des Sciences, Université Mohammed V-Agdal, Rabat, Morocco; 179 DSM/IRFU (Institut de Recherches sur les Lois Fondamentales de l’Univers), CEA Saclay (Commissariat à l’Energie Atomique et aux Energies Alternatives), Gif-sur-Yvette, France; 180 Santa Cruz Institute for Particle Physics, University of California Santa Cruz, Santa Cruz, CA USA; 181 Department of Physics, University of Washington, Seattle, WA USA; 182 Department of Physics and Astronomy, University of Sheffield, Sheffield, UK; 183 Department of Physics, Shinshu University, Nagano, Japan; 184 Fachbereich Physik, Universität Siegen, Siegen, Germany; 185 Department of Physics, Simon Fraser University, Burnaby, BC Canada; 186 SLAC National Accelerator Laboratory, Stanford, CA USA; 187 Faculty of Mathematics, Physics and Informatics, Comenius University, Bratislava, Slovak Republic; 188 Department of Subnuclear Physics, Institute of Experimental Physics of the Slovak Academy of Sciences, Kosice, Slovak Republic; 189 Department of Physics, University of Cape Town, Cape Town, South Africa; 190 Department of Physics, University of Johannesburg, Johannesburg, South Africa; 191 School of Physics, University of the Witwatersrand, Johannesburg, South Africa; 192 Department of Physics, Stockholm University, Stockholm, Sweden; 193 The Oskar Klein Centre, Stockholm, Sweden; 194 Physics Department, Royal Institute of Technology, Stockholm, Sweden; 195 Departments of Physics and Astronomy and Chemistry, Stony Brook University, Stony Brook, NY USA; 196 Department of Physics and Astronomy, University of Sussex, Brighton, UK; 197 School of Physics, University of Sydney, Sydney, Australia; 198 Institute of Physics, Academia Sinica, Taipei, Taiwan; 199 Department of Physics, Technion: Israel Institute of Technology, Haifa, Israel; 200 Raymond and Beverly Sackler School of Physics and Astronomy, Tel Aviv University, Tel Aviv, Israel; 201 Department of Physics, Aristotle University of Thessaloniki, Thessaloníki, Greece; 202 International Center for Elementary Particle Physics and Department of Physics, The University of Tokyo, Tokyo, Japan; 203 Graduate School of Science and Technology, Tokyo Metropolitan University, Tokyo, Japan; 204 Department of Physics, Tokyo Institute of Technology, Tokyo, Japan; 205 Department of Physics, University of Toronto, Toronto, ON Canada; 206 TRIUMF, Vancouver, BC Canada; 207 Department of Physics and Astronomy, York University, Toronto, ON Canada; 208 Faculty of Pure and Applied Sciences, University of Tsukuba, Tsukuba, Japan; 209 Department of Physics and Astronomy, Tufts University, Medford, MA USA; 210 Centro de Investigaciones, Universidad Antonio Narino, Bogotá, Colombia; 211 Department of Physics and Astronomy, University of California Irvine, Irvine, CA USA; 212 INFN Gruppo Collegato di Udine, Sezione di Trieste, Udine, Italy; 213 ICTP, Trieste, Italy; 214 Dipartimento di Chimica Fisica e Ambiente, Università di Udine, Udine, Italy; 215 Department of Physics, University of Illinois, Urbana, IL USA; 216 Department of Physics and Astronomy, University of Uppsala, Uppsala, Sweden; 217 Instituto de Física Corpuscular (IFIC) and Departamento de Física Atómica, Molecular y Nuclear and Departamento de Ingeniería Electrónica and Instituto de Microelectrónica de Barcelona (IMB-CNM), University of Valencia and CSIC, Valencia, Spain; 218 Department of Physics, University of British Columbia, Vancouver, BC Canada; 219 Department of Physics and Astronomy, University of Victoria, Victoria, BC Canada; 220 Department of Physics, University of Warwick, Coventry, UK; 221 Waseda University, Tokyo, Japan; 222 Department of Particle Physics, The Weizmann Institute of Science, Rehovot, Israel; 223 Department of Physics, University of Wisconsin, Madison, WI USA; 224 Fakultät für Physik und Astronomie, Julius-Maximilians-Universität, Würzburg, Germany; 225 Fachbereich C Physik, Bergische Universität Wuppertal, Wuppertal, Germany; 226 Department of Physics, Yale University, New Haven, CT USA; 227 Yerevan Physics Institute, Yerevan, Armenia; 228 Centre de Calcul de l’Institut National de Physique Nucléaire et de Physique des Particules (IN2P3), Villeurbanne, France; 229 CERN, Geneva, Switzerland

## Abstract

A search for single top-quark production via flavour-changing neutral current processes from gluon plus up- or charm-quark initial states in proton–proton collisions at the LHC is presented. Data collected with the ATLAS detector in 2012 at a centre-of-mass energy of 8 TeV and corresponding to an integrated luminosity of 20.3 fb$^{-1}$ are used. Candidate events for a top quark decaying into a lepton, a neutrino and a jet are selected and classified into signal- and background-like candidates using a neural network. No signal is observed and an upper limit on the production cross-section multiplied by the $t \rightarrow Wb$ branching fraction is set. The observed 95 % CL limit is $\sigma _{qg \rightarrow t} {\,\times \,} \mathcal {B}(t \rightarrow Wb)< {3.4}\,\mathrm{pb}$ and the expected 95 % CL limit is $\sigma _{qg \rightarrow t} \times \mathcal {B}(t \rightarrow Wb)< {2.9}\,\mathrm{pb}$. The observed limit can be interpreted as upper limits on the coupling constants of the flavour-changing neutral current interactions divided by the scale of new physics $\kappa _{ugt}/\Lambda < 5.8 \times 10^{-3}\, \mathrm{TeV}^{-1}$ and $\kappa _{cgt}/\Lambda < 13 \times 10^{-3}\, \mathrm{TeV}$ and on the branching fractions $\mathcal {B}(t \rightarrow ug) < {4.0 \times 10^{-5}}$ and $\mathcal {B}(t \rightarrow cg) < {20 \times 10^{-5}}$.

## Introduction

The top quark is the most massive elementary particle known, with a mass $m_{\text {top}}={173.3 \pm 0.8}\,\mathrm{GeV}$ [1] close to the electroweak symmetry breaking scale. This makes it an excellent object with which to test the Standard Model (SM) of particle physics, as well as to search for phenomena beyond the SM.

At the LHC, top quarks are primarily produced in pairs via the strong interaction. In addition to the predominant pair-production process, top quarks are produced singly through three different subprocesses via the weak interaction: the *t*-channel, which is the dominant process, involving the exchange of a space-like *W* boson; the *Wt* associated production, involving the production of a real *W* boson; and the *s*-channel process involving the production of a time-like *W* boson.

As a consequence of the large value, which is close to one, of the $V_{tb}$ element in the Cabibbo–Kobayashi–Maskawa (CKM) matrix, the predominant decay channel of top quarks is $t\rightarrow Wb$. Transitions between top quarks and other quark flavours mediated by neutral gauge bosons, so-called flavour-changing neutral currents (FCNC), are forbidden at tree level and suppressed at higher orders in the SM [2]. However, several extensions to the SM exist that significantly enhance the production rate and hence the branching fractions, $\mathcal {B}$, of FCNC processes. Examples of such extensions are the quark-singlet model [3–5], two-Higgs-doublet models with or without flavour conservation [6–11], the minimal supersymmetric standard model [12–18] or supersymmetry with *R*-parity violation [19, 20], models with extra quarks [21–23], or the topcolour-assisted technicolour model [24]. Reviews can be found in Refs. [25, 26]. Many of these models allow for enhanced FCNC production rates, e.g. by permitting FCNC interactions at tree level or introducing new particles in higher-order loop diagrams. The predicted branching fractions for top quarks decaying to a quark and a neutral boson can be as large as $10^{-5}$–$10^{-3}$ for certain regions of the parameter space in the models mentioned. However, the experimental limits have not excluded any specific extension of the SM for the process $t \rightarrow qg$ so far.

**Fig. 1:**
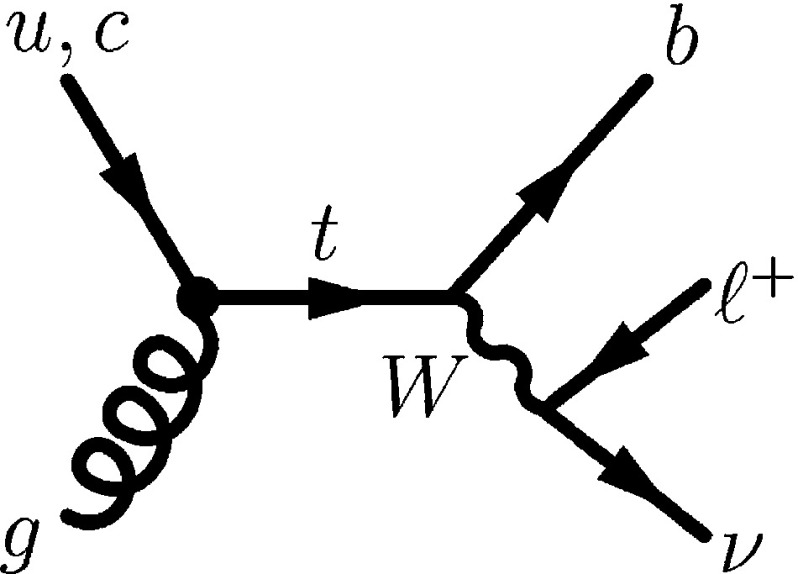
Leading-order Feynman diagram for FCNC top-quark production in the $qg \rightarrow t$ mode followed by the decay of the top quark into a *b*-quark and a *W* boson, where the *W* boson decays into a lepton and a neutrino

Among FCNC top-quark decays of the form $t \rightarrow qX$ with $X=Z,H,\gamma ,g$, modes involving a *Z* boson, a Higgs boson (*H*), or a photon ($\gamma$) are usually studied directly by searching for final states containing the corresponding decay particles. However, the mode $t \rightarrow qg$, where *q* denotes either an up quark, *u*, or a charm quark, *c*, is nearly indistinguishable from the overwhelming background of multi-jet production via quantum chromodynamic (QCD) processes. For the $t \rightarrow qg$ mode, much better sensitivity can be achieved by searching for anomalous single top-quark production ($qg\rightarrow t$) where a *u*- or *c*-quark and a gluon *g*, originating from the colliding protons, interact to produce a single top quark. A leading-order diagram for top-quark production in the $qg \rightarrow t$ mode as well as a SM decay of the top quark is shown in Fig. 1.^1^

Anomalous FCNC couplings can be described in a model-independent manner using an effective operator formalism [27], which assumes the SM to be the low-energy limit of a more general theory that is valid at very high energies. The effects of this theory below a lower energy scale, $\Lambda$, are perceived through a set of effective operators of dimension higher than four. The formalism therefore allows the new physics to be described by an effective Lagrangian consisting of the SM Lagrangian and a series of higher-dimension operators, which are suppressed by powers of $1/\Lambda$. The new physics scale, ${\Lambda }$, has a dimension of energy and is related to the mass cut-off scale above which the effective theory breaks down, hence characterising the energy scale at which the new physics manifests itself in the theory. A further method for simplifying the formalism is to only consider operators of interest that have no sizeable impact on physics below the TeV scale, following Ref. [28].

The interest of this paper lies in effective dimension-six operators, which contribute to flavour-changing interactions in the strong sector; thus no operators with electroweak gauge bosons are considered. In particular, the operators describing FCNC couplings to a single top quark are of interest here; they describe strong FCNC vertices of the form *qgt* and can be written as [29]:

$$\begin{aligned} \mathcal {O}_{uG\Phi }^{\,ij}=\bar{q}_{\mathrm L}^{\,i}\,\lambda ^a\,\sigma ^{\mu \nu }\,u_{\mathrm R}^j\,\tilde{\Phi }\,G^{a\mu \nu }\,, \end{aligned}$$

where $u_R^j$ stands for a right-handed quark singlet, $\bar{q}_{L}^{\,i}$ for a left-handed quark doublet, $G^{a\mu \nu }$ is the gluon field strength tensor, $\tilde{\Phi }$ the charge conjugate of the Higgs doublet, $\lambda ^a$ are the Gell-Mann matrices and $\sigma ^{\mu \nu }$ is the anti-symmetric tensor. The indices (*i*, *j*) of the spinors are flavour indices indicating the quark generation. By requiring a single top quark in the interaction, one of the indices can always be set equal to 3 while the other index is either 1 or 2. Hence, the remaining fermion field in the interaction is either a *u*- or a *c*-quark. Apart from direct single top-quark production, these operators give rise to interactions of the form $gg \rightarrow tq$ and $gq \rightarrow tg$. The processes considered are a subset of these, where a *u*-quark, *c*-quark or gluon originating from the colliding protons interacts through an *s*-, *t*- or *u*-channel process to produce a single top quark, either via a $(2 \rightarrow 2)$ process or without the associated production of additional gluons or light quarks via a $(2 \rightarrow 1)$ process.

The corresponding strong FCNC Lagrangian usually is written as [29]:

$$\begin{aligned} \mathcal {L}_{\mathrm S} = -g_{\mathrm s} \sum _{q=u,c}\,\frac{\kappa _{qgt}}{\Lambda }\,\bar{q}\,\lambda ^a\,\sigma ^{\mu \nu }\,(f_{q} + h_{q}\gamma _5)\,t\,G^{a}_{\mu \nu } + \text {h.c.}\,, \end{aligned}$$

with the real and positive parameters $\kappa _{gqt}\,(q=u,c)$ that relate the strength of the new couplings to the strong coupling strength, $g_{\mathrm s}$, and where *t* denotes the top-quark field. The parameters $f_{q}$ and $h_{q}$ are real, vector and axial chiral parameters, respectively, which satisfy the relation $|f_{q}|^2 + |h_{q}|^2 = 1$. This Lagrangian contributes to both the production and decay of top quarks.

Experimental limits on the branching fractions of the FCNC top-quark decay channels have been set by experiments at the LEP, HERA, Tevatron and LHC accelerators. At present the most stringent upper limits at 95 % confidence level (CL) for the coupling constants $\kappa _{\gamma qt}$ and $\kappa _{qgt}$ are $\kappa _{\gamma qt}/m_{\text {top}}< {0.12}\,\mathrm{GeV^{-1}}$ [30] (ZEUS, HERA) and $\mathcal {B}(t\rightarrow qg) < {5.7 \times 10^{-5}}$ (*ugt*) and $\mathcal {B}(t\rightarrow qg) < {2.7 \times 10^{-4}}$ (*cgt*) [31] (ATLAS, LHC). In the case of $t \rightarrow qZ$, upper limits on the branching fractions of the top-quark decay have been determined to be $\mathcal {B}(t\rightarrow qZ) < {0.05}\,{\%}$ [32] (CMS, LHC). Finally, the most stringent limit for the decay $t\rightarrow qH$ is measured to be $\mathcal {B}(t\rightarrow qH) < {0.79}\,{\%}$ [33] (ATLAS, LHC).

In the allowed region of parameter space for $\kappa _{qgt}/\Lambda$, the FCNC production cross-section for single top quarks is of the order of picobarns, while the branching fraction for FCNC decays is very small, i.e. below 1 %. Top quarks are therefore reconstructed in the SM decay mode $t\rightarrow Wb$. The *W* boson can decay into a quark–antiquark pair ($W\rightarrow q_1 \bar{q}_{2}$) or a charged lepton–neutrino pair ($W\rightarrow \ell \nu$); only the latter is considered here. This search targets the signature from the $qg \rightarrow t \rightarrow W(\rightarrow \ell \nu )\,b$ process. Events are characterised by an isolated high-energy charged lepton (electron or muon), missing transverse momentum from the neutrino and exactly one jet produced by the hadronisation of the *b*-quark. Events with a *W* boson decaying into a $\tau$ lepton, where the $\tau$ decays into an electron or a muon, are also included. Several SM processes have the same final-state topology and are considered as background to the FCNC analysis. The main backgrounds are *V*+jets production (especially in association with heavy quarks), where *V* denotes a *W* or a *Z* boson, SM top-quark production, diboson production, and multi-jet production via QCD processes. The studied process can be differentiated from SM single top-quark production, which is usually accompanied by additional jets. Furthermore, FCNC production has kinematic differences from the background processes, such as lower transverse momenta of the top quark.

This paper is organised as follows: Sect. 2 provides a description of the ATLAS detector. Section 3 gives an overview of the data and Monte Carlo (MC) samples used for the simulation of signal and expected background events from SM processes. In Sect. 4 the event selection is presented. The methods of event classification into signal- and background-like events using a neural network are discussed in Sect. 5 and sources of systematic uncertainty are summarised in Sect. 6. The results are presented in Sect. 7 and the conclusions are given in Sect. 8.

## ATLAS detector

The ATLAS detector [34] is a multipurpose collider detector built from a set of sub-detectors, which cover almost the full solid angle around the interaction point.^2^ It is composed of an inner tracking detector (ID) close to the interaction point surrounded by a superconducting solenoid providing a 2T axial magnetic field, electromagnetic and hadronic calorimeters, and a muon spectrometer (MS). The ID consists of a silicon pixel detector, a silicon microstrip detector providing tracking information within pseudorapidity $|\eta | < 2.5$, and a straw-tube transition radiation tracker that covers $|\eta | < 2.0$. The central electromagnetic calorimeter is a lead and liquid-argon (LAr) sampling calorimeter with high granularity, and is divided into a barrel region that covers $|\eta | < 1.475$ and endcap regions that cover $1.375 < |\eta | < 3.2$. An iron/scintillator tile calorimeter provides hadronic energy measurements in the central pseudorapidity range. The endcap and forward regions are instrumented with LAr calorimeters for both the electromagnetic and hadronic energy measurements, and extend the coverage to $|\eta | = 4.9$. The MS covers $|\eta | < 2.7$ and consists of three large superconducting toroids with eight coils each, a system of trigger chambers, and precision tracking chambers.

## Data and simulated samples

This analysis is performed using $\sqrt{s}= {8} \, \mathrm{TeV}$ proton–proton (*pp*) collision data recorded by the ATLAS experiment in 2012. Stringent detector and data quality requirements are applied, resulting in a data sample with a total integrated luminosity of 20.3 ${{\rm fb}^{-1}}$.

### Trigger requirements

ATLAS employs a three-level trigger system for selecting events to be recorded. The first level (L1) is built from custom-made hardware, while the second and third levels are software based and collectively referred to as the high-level trigger (HLT). The datasets used in this analysis are defined by high-$p_{\text {T}}$ single-electron or single-muon triggers [35, 36].

For the L1 calorimeter trigger, which is based on reduced calorimetric information, a cluster in the electromagnetic calorimeter is required with $E_{\text {T}}> {30}\,\mathrm{GeV}$ or with $E_{\text {T}}> {18}\,\mathrm{GeV}$. The energy deposit must be well separated from other clusters. At the HLT, the full granularity of the calorimeter and tracking information is available. The calorimeter cluster is matched to a track and the trigger electron candidate is required to have $E_{\text {T}}> {60}\,\mathrm{GeV}$ or $E_{\text {T}}> {24}\,\mathrm{GeV}$ with additional isolation requirements.

The single-muon trigger is based on muon candidates reconstructed in the MS. The triggered events require a L1 muon trigger-chamber track with a 15 GeV threshold on the $p_{\text {T}}$ of the track. At the HLT, the requirement is tightened to $p_{\text {T}}> {24}\,\mathrm{GeV}$ with, or 36 GeV without, an isolation criterion.

### Simulated events

Simulated event samples are used to evaluate signal and background efficiencies and uncertainties as well as to model signal and background shapes.

For the direct production of top quarks via FCNC, MEtop [29] is used for simulating strong FCNC processes at next-to-leading order (NLO) in QCD. It introduces strong top-quark FCNC interactions through effective operators. By comparing kinematic distributions for different FCNC couplings, it has been verified that the kinematics of the signal process are independent of the a priori unknown FCNC coupling strength. As a conservative approach, only left-handed top quarks (as in the SM) are produced, and the decay of the top quark is assumed also to be as in the SM.^3^ The CT10 [37] parton distribution function (PDF) sets are used for the generation of the signal events and the renormalisation and factorisation scales are set to the top-quark mass.

The Powheg-box [38] generator with the CT10 PDF sets is used to generate $t\bar{t}$ [39] and electroweak single top-quark production in the *t*-channel [40], *s*-channel [41] and *Wt*-channel [42]. All processes involving top quarks, including the strong FCNC processes, are produced assuming $m_{\text {top}}= {172.5}\,\mathrm{GeV}$. The parton shower and the underlying event are added using Pythia 6.426 [43], where the parameters controlling the modelling are set to the values of the Perugia 2011C tune [44].

Vector-boson production in association with jets (*V*+jets) is simulated using the multi-leg leading-order (LO) generator Sherpa 1.4.1 [45] with its own parameter tune and the CT10 PDF sets. Sherpa is used not only to generate the hard process, but also for the parton shower and the modelling of the underlying event. *W*+jets and *Z*+jets events with up to five additional partons are generated. The CKKW method [46] is used to remove overlap between partonic configurations generated by the matrix element and by parton shower evolution. Double counting between the inclusive *V*+*n* parton samples and samples with associated heavy-quark pair production is avoided consistently by using massive *c*- and *b*-quarks in the shower.

Diboson events (*WW*, *WZ* and *ZZ*) are produced using Alpgen 2.14 [47] and the CTEQ6L1 PDF sets [48]. The partonic events are showered with Herwig 6.5.20 [49], and the underlying event is simulated with the Jimmy 4.31 [50] model using the ATLAS Underlying Event Tune 2 [51].

All the generated samples are passed through the full simulation of the ATLAS detector [52] based on Geant4 [53] and are then reconstructed using the same procedure as for data. The simulation includes the effect of multiple *pp* collisions per bunch crossing. The events are weighted such that the average distribution of the number of collisions per bunch crossing is the same as in data. In addition, scale factors are applied to the simulated events to take into account small differences observed between the efficiencies for the trigger, lepton identification and *b*-quark jet identification. These scale factors are determined using control samples.

## Event selection

The expected signature of signal events is used to perform the event selection. Events containing exactly one isolated electron or muon, missing transverse momentum and one jet, which is required to be identified as a jet originating from a *b*-quark, are selected.

### Object definition and event selection

Electron candidates are selected from energy deposits (clusters) in the LAr electromagnetic calorimeter associated with a well-measured track fulfilling strict quality requirements [54]. Electron candidates are required to satisfy $p_{\text {T}}> {25}\,\mathrm{GeV}$ and $|\eta _{\text {clus}}| < 2.47$, where $\eta _{\text {clus}}$ denotes the pseudorapidity of the cluster. Clusters falling in the calorimeter barrel–endcap transition region, corresponding to $1.37<|\eta _{\text {clus}}|<1.52$, are ignored. High-$p_{\text {T}}$ electrons associated with the *W*-boson decay can be mimicked by hadronic jets reconstructed as electrons, electrons from the decay of heavy quarks, and photon conversions. Since electrons from the *W*-boson decay are typically isolated from hadronic jet activity, backgrounds can be suppressed by isolation criteria, which require minimal calorimeter activity and only allow low-$p_{\text {T}}$ tracks in an $\eta$–$\phi$ cone around the electron candidate. Isolation cuts are optimised to achieve a uniform cut efficiency of 90 % as a function of $\eta _{\text {clus}}$ and transverse energy, $E_{\text {T}}$. The direction of the electron candidate is taken as that of the associated track. For the calorimeter isolation a cone size of $\Delta R = 0.2$ is used. In addition, the scalar sum of all track transverse momenta within a cone of size $\Delta R = 0.3$ around the electron direction is required to be below a $p_{\text {T}}$-dependent threshold in the range between 0.9 and 2.5 GeV. The track belonging to the electron candidate is excluded from this requirement.

Muon candidates are reconstructed by matching track segments or complete tracks in the MS with tracks found in the ID [55]. The final candidates are required to have a transverse momentum $p_{\text {T}}> {25}\,\mathrm{GeV}$ and to be in the pseudorapidity region $|\eta |<2.5$. Isolation criteria are applied to reduce background events in which a high-$p_{\text {T}}$ muon is produced in the decay of a heavy-flavour quark. An isolation variable [56] is defined as the scalar sum of the transverse momenta of all tracks with $p_{\text {T}}$ above 1 GeV, except the one matched to the muon, within a cone of size $\Delta R_{\text {iso}} = {10}\,\mathrm{GeV}/p_{\text {T}}(\mu )$. Muon candidates are accepted if they have an isolation to $p_{\text {T}}(\mu )$ ratio of less than 0.05. An overlap removal is applied between the electrons and the muons, rejecting the event if the electron and the muon share the same ID track.

Jets are reconstructed using the anti-$k_{t}$ algorithm [57] with a radius parameter of 0.4, using topological clusters [58] as inputs to the jet finding. The clusters are calibrated with a local cluster weighting method [59]. Calibrated jets using an energy- and $\eta$-dependent simulation-based calibration scheme, with in situ corrections based on data, are at first required to have $p_{\text {T}}> {25}\,\mathrm{GeV}$ and $|\eta |<2.5$. The jet energy is further corrected for the effect of multiple *pp* interactions, both in data and in simulated events.

If any jet is within $\Delta R = 0.2$ of an electron, the closest jet is removed, since in these cases the jet and the electron are very likely to correspond to the same physics object. Remaining electron candidates overlapping with jets within a distance $\Delta R<0.4$ are subsequently rejected. To reject jets from pile-up events, a so-called jet-vertex fraction criterion is applied for jets with $p_{\text {T}}< {50}\,\mathrm{GeV}$ and $|\eta | <2.4$: at least 50 % of the scalar sum of the $p_{\text {T}}$ of tracks within a jet is required to be from tracks compatible with the primary vertex^4^ associated with the hard-scattering collision. The final selected jet is required to have $p_{\text {T}}> {30}\,\mathrm{GeV}$ and must also be identified as a jet originating from a *b*-quark (*b*-tagged).

In this analysis, a *b*-tagging algorithm that is optimised to improve the rejection of *c*-quark jets is used, since $W+c$ production is a major background. A neural-network-based algorithm is used, which combines three different algorithms exploiting the properties of a *b*-hadron decay in a jet [60]. The chosen working point corresponds to a *b*-tagging efficiency of 50 %, when cutting on the discriminant, and a *c*-quark jet and light-parton jet mistag acceptance of 3.9 and 0.07 %, respectively, as measured in $t\bar{t}$ events [61, 62].

The missing transverse momentum (with magnitude $E_{\text {T}}^{\text {miss}}$) is calculated based on the vector sum of energy deposits in the calorimeter projected onto the transverse plane [63]. All cluster energies are corrected using the local cluster calibration scheme. Clusters associated with a high-$p_{\text {T}}$ jet or electron are further calibrated using their respective energy corrections. In addition, contributions from the $p_{\text {T}}$ of selected muons are included in the calculation of $E_{\text {T}}^{\text {miss}}$. Due to the presence of a neutrino in the final state of the signal process, $E_{\text {T}}^{\text {miss}}> {30}\,\mathrm{GeV}$ is required. Lepton candidates in multi-jet events typically arise from charged tracks being misidentified as leptons, electrons arising from converted photons and leptons from *c*- and *b*-hadron decays. Such candidates are collectively referred to as fake leptons. As such, the multi-jet events tend to have low $E_{\text {T}}^{\text {miss}}$ and low *W*-boson transverse mass,^5^ $m_{\text {T}}(W)$, relative to single top-quark events. Therefore, an additional requirement on $m_{\text {T}}(W)$ is an effective way to reduce this background. The selection applied is $m_{\text {T}}(W)> {50}\,\mathrm{GeV}$. In order to further suppress the multi-jet background and also to remove poorly reconstructed leptons with low transverse momentum, a requirement on the transverse momentum of leptons and the azimuthal angle between the lepton and jet is applied:

1 $$\begin{aligned} p_{\text {T}}^{\ell } > {90}\,\mathrm{GeV} \left( 1- \frac{\pi - |\Delta \phi (\ell , \text {jet})|}{\pi -2}\right) \,. \end{aligned}$$

**Fig. 2:**
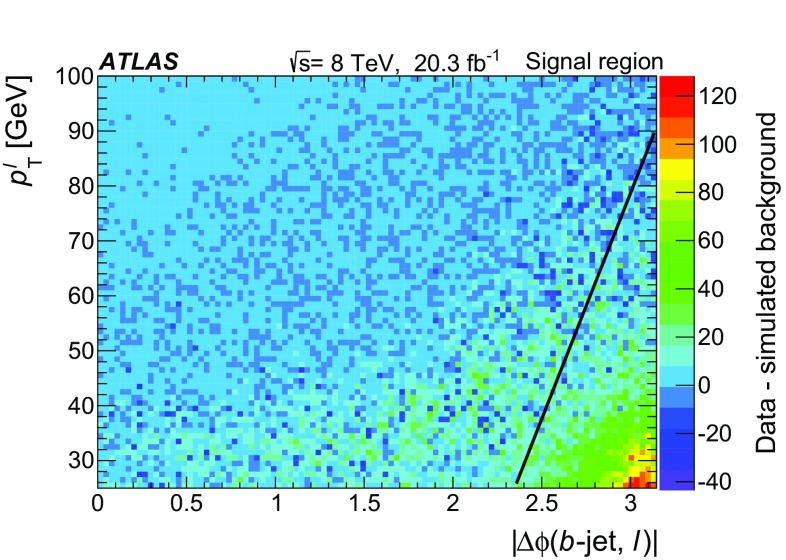
The transverse momentum of the lepton versus the azimuthal angle between the lepton and the jet. The *colours* indicate the number of events in data after the simulated backgrounds except the multi-jet contribution have been subtracted and before the cut given by Eq. 1 is applied. The *solid black line* shows the cut

The parameters of the cut are motivated by the distribution of multi-jet events, obtained in the signal region, where the simulated backgrounds except the multi-jet contribution are subtracted from data. Almost no signal events are removed by this cut. The distribution of the transverse momentum of the lepton versus the azimuthal angle between the lepton and the jet is shown in Fig. 2.

In addition to the signal region defined by this selection, a control region is defined with the same kinematic requirements, but with a less stringent *b*-tagging requirement with an efficiency of 85 %, and excluding events passing the tighter signal-region *b*-tagging selection. This control region is designed such that the resulting sample is dominated by *W*+jets production, which is the dominant background.

### Background estimation

For all background processes except the multi-jet background, the normalisations are estimated by using Monte Carlo simulation scaled to the theoretical cross-section predictions, using $m_{\text {top}}= {172.5}\,\mathrm{GeV}$. In order to check the modelling of kinematic distributions, correction factors to the normalisation of the *W*+jets and $t\bar{t}$ and single-top processes are subsequently determined simultaneously in the context of the multi-jet background estimation.

The SM single top-quark production cross-sections are calculated to approximate next-to-next-to-leading-order (NNLO) precision. The production via the *t*-channel exchange of a virtual *W* boson has a predicted cross-section of 87 pb [64]. The cross-section for the associated production of an on-shell *W* boson and a top quark (*Wt* channel) has a predicted value of 22.3 pb [65], while the *s*-channel production has a predicted cross-section of 5.6 pb [66]. The resulting weighted average of the theoretical uncertainties including PDF and scale uncertainties of these three processes is 10 %.

The cross-section of the $t\bar{t}$ process is normalised to 238 pb, calculated at NNLO in QCD including resummation of next-to-next-to-leading logarithmic (NNLL) soft gluon terms [67–71] with Top++2.0 [72]. The PDF and $\alpha _{\mathrm {s}}$ uncertainties are calculated using the PDF4LHC prescription [73] with the MSTW2008 NNLO [74, 75] at 68 % $\text {CL}$, the CT10 NNLO [37, 76], and the NNPDF 2.3 [77] PDF sets, and are added in quadrature to the scale uncertainty, yielding a final uncertainty of 6 %.

The cross-sections for inclusive *W*- and *Z*-boson production are predicted with NNLO precision using the FEWZ program [78, 79], resulting in a LO-to-NNLO *K*-factor of 1.10 and an uncertainty of 4 %. The uncertainty includes the uncertainty on the PDF and scale variations. The scale factor is applied to the prediction based on the LO Sherpacalculation and the flavour composition is also taken from the MC samples. The modelling of the transverse momentum of the *W* boson in the *W*+jets sample is improved by reweighting the simulated samples to data in the *W*+jets-dominated control region.

LO-to-NLO *K*-factors obtained with MCFM [80] of the order of 1.3 are applied to the Alpgen LO predictions for diboson production. Since the diboson process is treated together with *Z*-boson production in the statistical analysis and the fraction of selected events is only 5 %, the same uncertainties as used for the *Z*+jets process are assumed.

Multi-jet events may be selected if a jet is misidentified as an isolated lepton or if the event has a non-prompt lepton that appears to be isolated. The normalisation of this background is obtained from a fit to the observed $E_{\text {T}}^{\text {miss}}$ distribution, performed both in the signal and control regions. In order to construct a sample of multi-jet background events, different methods are adopted for the electron and muon channels. The ‘jet-lepton’ model is used in the electron channel while the ‘anti-muon’ model is used in the muon channel [81]. In the jet-lepton model, a shape for the multi-jet background is established using events from a Pythia dijet sample, which are selected using same criteria as the standard selection, but with a jet used in place of the electron candidate. Each candidate jet has to fulfil the same $p_{\text {T}}$ and $\eta$ requirements as a standard lepton and deposit 80–95 % of its energy in the electromagnetic calorimeter. Events with an electron candidate passing the electron cuts described in Sect. 4.1 are rejected and an event is accepted if exactly one ’jet-lepton’ is found. The anti-muon model is derived from collision data. In order to select a sample that is highly enriched with muons from multi-jet events, some of the muon identification cuts are inverted or changed, e.g. the isolation criteria are inverted.

To determine the normalisation of the multi-jet background template, a binned maximum-likelihood fit is performed on the $E_{\text {T}}^{\text {miss}}$ distribution using the observed data, after applying all selection criteria except for the cut on $E_{\text {T}}^{\text {miss}}$. Fits are performed separately in two $\eta$ regions for electrons: in the endcap ($|\eta | > 1.52$) and central ($|\eta | < 1.37$) region of the electromagnetic calorimeter, i.e. the transition region is excluded. For muons, the complete $\eta$ region is used. The multi-jet templates for both the electrons and the muons are fitted together with templates derived from MC simulation for all other background processes (top quark, *W*+light flavour (LF), *W*+heavy flavour (HF), *Z*+jets, dibosons). Acceptance uncertainties are accounted for in the fitting process in the form of additional constrained nuisance parameters. For the purpose of these fits, the contributions from $W$+LF and $W$+HF, the contributions from $t\bar{t}$ and single top-quark production, and the contributions from *Z*+jets and diboson production are each combined into one template. The normalisation of the template for *Z*+jets and diboson production is fixed during the fit, as its contribution is very small.

**Fig. 3:**
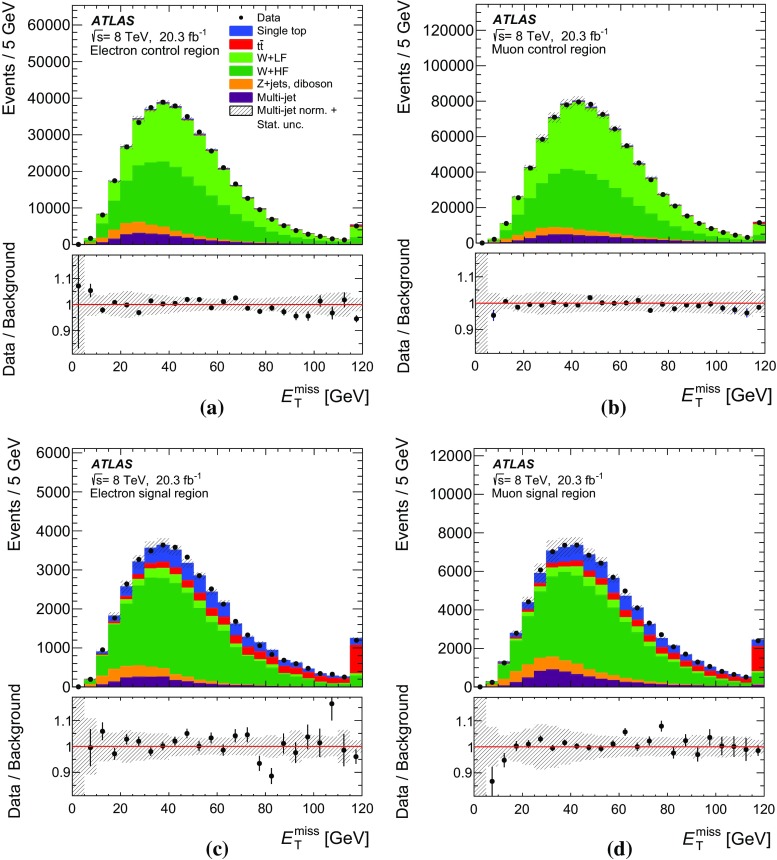
Fitted distributions of the missing transverse momentum $E_{\text {T}}^{\text {miss}}$ for **a** central electrons and **b** muons in the control region and for **c** central electrons and **d** muons in the signal region. The last histogram bin includes overflow events and the *hatched error bands* contain the MC statistical uncertainty combined with the normalisation uncertainty on the multi-jet background

The $E_{\text {T}}^{\text {miss}}$ distributions after rescaling the different backgrounds and the multi-jets template to their respective fit results are shown in Fig. 3 for both the electron and the muon channels. The fitted scale factors for the other templates are close to 1.

Table 1 provides the event yields after the complete event selection for the control and signal regions. The yields are calculated using the acceptance from MC samples normalised to their respective theoretical cross-sections including the (N)NLO *K*-factors, while the number of expected events for the multi-jet background is obtained from the maximum-likelihood fit. Each event yield uncertainty combines the statistical uncertainty, originating from the limited size of the simulation samples, with the uncertainty on the cross-section or normalisation. The observed event yield in data agrees well with the background prediction. For comparison, a 1 pb FCNC cross-section would lead to 530 events in the signal region. The corresponding efficiency for selecting FCNC events is 3.1 %.

**Table 1:** Number of observed and expected events in the control and signal region for all lepton categories added together. The uncertainties shown are derived using the statistical uncertainty from the limited size of the samples and the uncertainty on the theoretical cross-section only or multi-jet normalisation. The scale factors obtained from the multi-jet background fit are not applied when determining the expected number of events

Process	Control region	Signal region
Single top	11,500 $\pm$ 620	14,400 $\pm$ 770
$t\bar{t}$	10,700 $\pm$ 650	12,000 $\pm$ 740
*W*+LF	526,900 $\pm$ 130,000	6700 $\pm$ 1900
*W*+HF	445,200 $\pm$ 240,000	62,100 $\pm$ 34,000
*Z*+jets	40,000 $\pm$ 9700	4990 $\pm$ 1200
Multi-jet	68,300 $\pm$ 12,000	7430 $\pm$ 1300
Total expected	1,100,000 $\pm$ 280,000	107,000 $\pm$ 34,000
Data	1,112,225	108,152

**Fig. 4:**
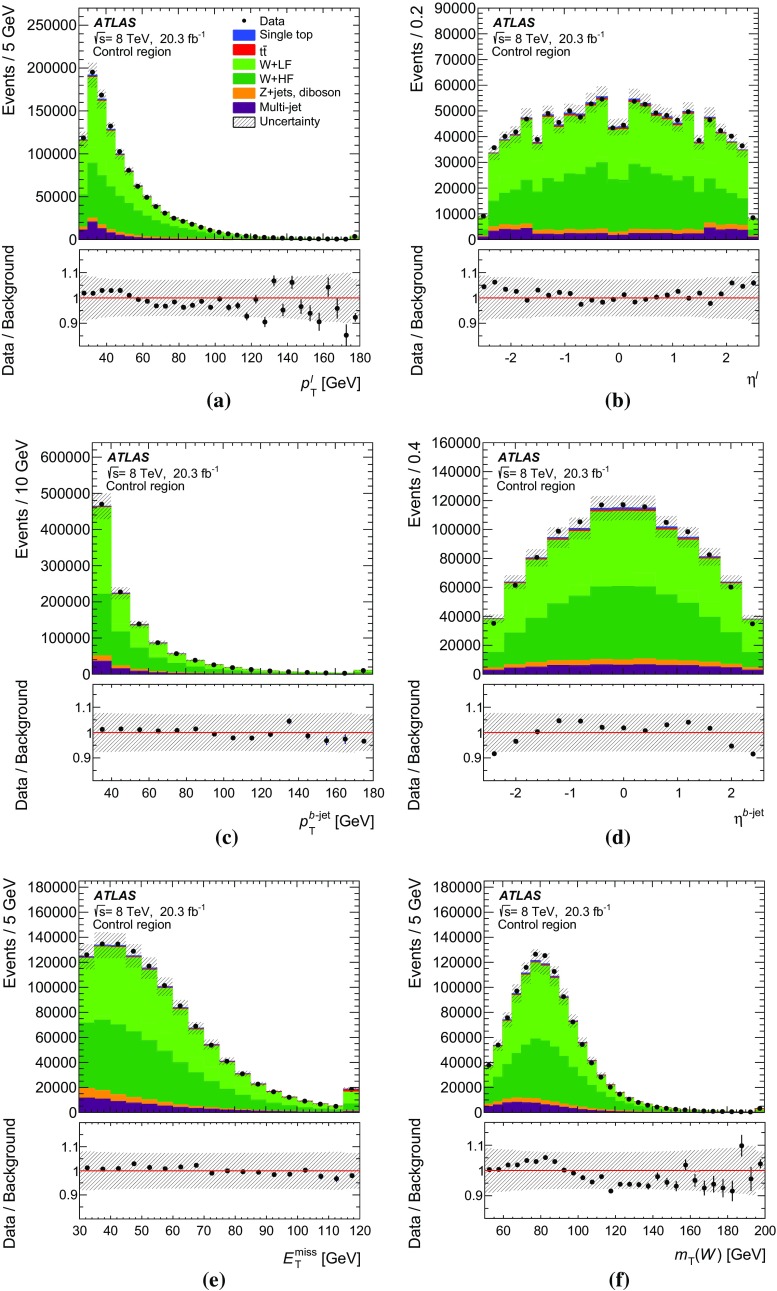
Kinematic distributions in the control region for the combined electron and muon channels. All processes are normalised to the result of the binned maximum-likelihood fit used to determine the fraction of multi-jet events. Shown are: **a** the transverse momentum and **b** pseudorapidity of the lepton, **c** the transverse momentum and **d** pseudorapidity of the jet, **e** the missing transverse momentum and **d** *W*-boson transverse mass. The last histogram bin includes overflow events and the *hatched band* indicates the combined statistical and systematic uncertainties, evaluated after the fit discussed in Sect. 7

Kinematic distributions in the control region of the identified lepton, reconstructed jet, $E_{\text {T}}^{\text {miss}}$and $m_{\text {T}}(W)$ are shown in Fig. 4 for the combined electron and muon channels. These distributions are normalised using the scale factors obtained in the $E_{\text {T}}^{\text {miss}}$ fit to estimate the multi-jet background. Overall, good agreement between the observed and expected distributions is seen. The trends that can be seen in some of the distributions are covered by the systematic uncertainties.

## Analysis strategy

As no single variable provides sufficient discrimination between signal and background events and the separation power is distributed over many correlated variables, multivariate analysis techniques are necessary to separate signal candidates from background candidates. A neural-network (NN) classifier [82] that combines a three-layer feed-forward neural network with a preprocessing of the input variables is used. The network infrastructure consists of one input node for each input variable plus one bias node, an arbitrary number of hidden nodes, and one output node, which gives a continuous output in the interval $[-1,1]$. The training is performed with a mixture of 50 % signal and 50 % background events, where the different background processes are weighted according to their number of expected events. Only processes from simulated events are considered in the training, i.e. no multi-jet events are used. In order to check that the neural network is not overtrained, 20 % of the available simulated events are used as a test sample. Subsequently, the NN classifier is applied to all samples.

**Table 2:** Variables used in the training of the neural network ordered by their descending importance

Variable	Definition
$m_{\text {T}}\,(\text {top})$	Transverse mass of the reconstructed top quark
$p_{\text {T}}^{\ell }$	Transverse momentum of the charged lepton
$\Delta R\,(\text {top},\ell )$	Distance in the $\eta$–$\phi$ plane between the reconstructed top quark and the charged lepton
$p_{\text {T}}^{b\text {-jet}}$	Transverse momentum of the *b*-tagged jet
$\Delta \phi \,(\text {top},b\text {-jet})$	Difference in azimuth between the reconstructed top quark and the *b*-tagged jet
$\cos \theta \,(\ell ,b\text {-jet})$	Opening angle of the three-vectors between the charged lepton and the *b*-tagged jet
$q^{\ell }$	Charge of the lepton
$m_{\text {T}}(W)$	*W*-boson transverse mass
$\eta ^{\ell }$	Pseudorapidity of the charged lepton
$\Delta \phi \,(\text {top},W)$	Difference in azimuth between the reconstructed top quark and the *W* boson
$\Delta R\,(\text {top},b\text {-jet})$	Distance in the $\eta$–$\phi$ plane between the reconstructed top quark and the *b*-tagged jet
$\eta ^{\text {top}}$	Pseudorapidity of the reconstructed top quark
$p_{\text {T}}^{W}$	Transverse momentum of the *W* boson

The $qg\rightarrow t\rightarrow b\ell \nu$ process is characterised by three main differences from SM processes. Firstly, the $p_{\text {T}}$ distribution of the top quark is much softer than the $p_{\text {T}}$ distribution of top quarks produced through SM top-quark production, since the top quark is produced almost without transverse momentum. Hence, the *W* boson and *b*-quark from the top-quark decay are produced almost back-to-back in the transverse plane. Secondly, unlike in the *W* / *Z*+jets and diboson backgrounds, the *W* boson from the top-quark decay has a high momentum and its decay products tend to have small angles. Lastly, the top-quark charge asymmetry differs between FCNC processes and SM processes in the *ugt* channel. In *pp* collisions, the FCNC processes are predicted to produce four times more single top quarks than anti-top quarks, whereas in SM single top-quark production and in all other SM backgrounds this ratio is at most two. Several categories of variables are considered as potential discriminators between the signal and background processes. Apart from basic event kinematics such as the $m_{\text {T}}(W)$ or $H_{\text {T}}$ (the scalar sum of the transverse momenta of all objects in the final state), various object combinations are considered as well. These include the basic kinematic properties of reconstructed objects like the *W* boson and the top quark, as well as angular distances in $\eta$ and $\phi$ between the reconstructed and final-state objects in the laboratory frame and in the rest frames of the *W* boson and the top quark. In order to reconstruct the four-vector of the *W* boson, a mass constraint is used. A detailed description of the top-quark reconstruction is given in Ref. [83]. Further, integer variables such as the charge of the lepton are considered.

**Fig. 5:**
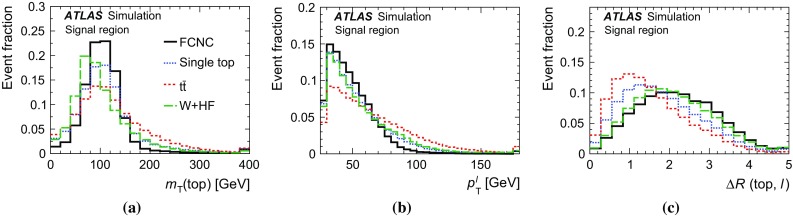
Probability densities of the three most important discriminating variables: **a** the transverse mass of the reconstructed top quark; **b** the transverse momentum of the charged lepton; and **c** the distance in the $\eta$–$\phi$ plane between the charged lepton and the reconstructed top quark. The last histogram bin includes overflows

The ranking of the variables in terms of their discrimination power is automatically determined as part of the preprocessing step and is independent of the training procedure [84].^6^ Only the highest-ranking variables are chosen for the training of the neural network. Each variable is tested beforehand for agreement between the background model and the distribution of the observed events in the control region. Using only variables with an a priori defined separation power, 13 variables remain in the network. Table 2 shows a summary of the variables used, ordered by their importance. The probability density of the three most important discriminating variables for the dominant background processes together with the signal is displayed in Fig. 5.

**Fig. 6:**
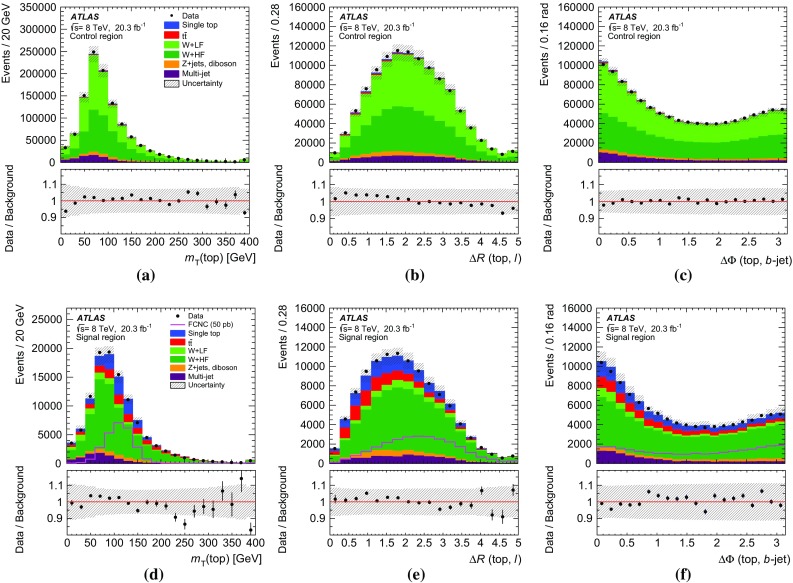
Distributions of three important discriminating variables (except for the transverse momentum of the lepton): **a**, **d** the top-quark transverse mass in the control and signal regions; **b**, **e** the $\Delta R$ between the lepton and the reconstructed top quark in the control and signal regions; **c**, **f** the $\Delta \phi$ between the jet and the reconstructed top quark. All processes are normalised using the scale factors obtained in the binned maximum-likelihood fit to the $E_{\text {T}}^{\text {miss}}$ distribution. The FCNC signal cross-section is scaled to 50 pb and overlayed on the distributions in the signal region. The last histogram bin includes overflow events and the *hatched band* indicates the combined statistical and systematic uncertainties, evaluated after the fit discussed in Sect. 7

**Fig. 7:**
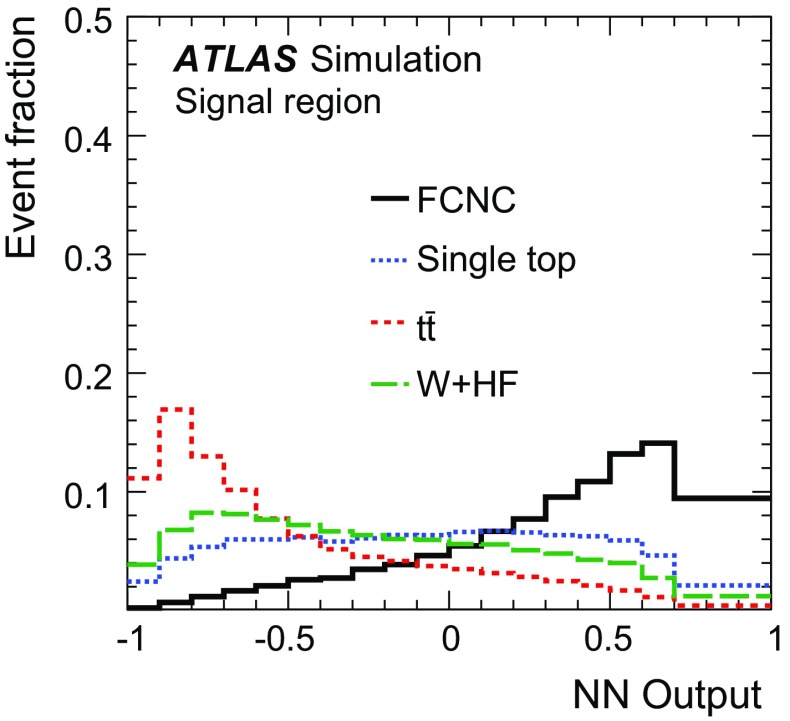
Probability density of the neural-network output distribution for the signal and the most important background processes

The distributions for three of the four most important variables in the control and signal regions are shown in Fig. 6. The shape of the multi-jet background is obtained using the samples described in Sect. 4.2. The distribution of $p_{\text {T}}^{\ell }$ is shown in Fig. 7 a for the control region. The distributions are normalised using the scale factors obtained in the binned maximum-likelihood fit to the $E_{\text {T}}^{\text {miss}}$ distribution.

**Fig. 8:**
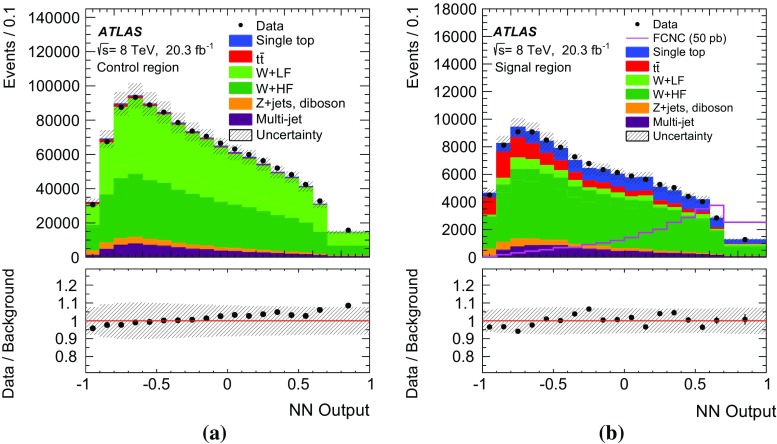
Neural-network output distribution **a** in the control region and **b** in the signal region. The shape of the signal scaled to 50 pb is shown in **b**. All background processes are shown normalised to the result of the binned maximum-likelihood fit used to determine the fraction of multi-jet events. The *hatched band* indicates the combined statistical and systematic uncertainties, evaluated after the fit discussed in Sect. 7

The resulting neural-network output distributions for the most important background processes and the signal are displayed in Fig. 7 as probability densities and in Fig. 8a, b normalised to the number of expected events in the control and signal regions, respectively. Signal-like events have output values close to 1, whereas background-like events accumulate near $-1$. Overall, good agreement within systematic uncertainties between data and the background processes is observed in both the control and signal regions.

## Systematic uncertainties

Systematic uncertainties are assigned to account for detector calibration and resolution uncertainties, as well as the uncertainties on theoretical predictions. These can affect the normalisation of the individual backgrounds and the signal acceptance (acceptance uncertainties) as well as the shape of the neural-network output distribution (shape uncertainties). Quoted relative uncertainties refer to acceptance of the respective processes unless stated otherwise.

### Object modelling

The effects of the systematic uncertainties due to the residual differences between data and Monte Carlo simulation, uncertainties on jets, electron and muon reconstruction after calibration, and uncertainties on scale factors that are applied to the simulation are estimated using pseudo-experiments.

Uncertainties on the muon (electron) trigger, reconstruction and selection efficiency scale factors are estimated in measurements of $Z \rightarrow \mu \mu$ ($Z \rightarrow e e$ and $W \rightarrow e\nu$) production. The scale factor uncertainties are as large as 5 %. To evaluate uncertainties on the lepton momentum scale and resolution, the same processes are used [85]. The uncertainty on the charge misidentification acceptances were studied and found to be negligible for this analysis.

The jet energy scale (JES) is derived using information from test-beam data, LHC collision data and simulation. Its uncertainty varies between 2.5 and 8 %, depending on jet $p_{\text {T}}$ and $\eta$ [59]. This includes uncertainties in the fraction of jets induced by gluons and mismeasurements due to close-by jets. Additional uncertainties due to pile-up can be as large as 5 %. An additional jet energy scale uncertainty of up to 2.5 %, depending on the $p_{\text {T}}$ of the jet, is applied for *b*-quark-induced jets due to differences between light-quark and gluon jets compared to jets containing *b*-hadrons. Additional uncertainties are from the modelling of the jet energy resolution and the missing transverse momentum, which accounts for contributions of calorimeter cells not matched to any jets, soft jets, and pile-up. The effect of uncertainties associated with the jet-vertex fraction is also considered for each jet.

Since the analysis makes use of *b*-tagging, the uncertainties on the *b*- and *c*-tagging efficiencies and the mistag acceptance [61, 62] are taken into account.

### Multi-jet background

For the multi-jet background, an uncertainty on the estimated multi-jet fractions and the modelling is included. The systematic uncertainty on the fractions, as well as a shape uncertainty, are obtained by comparing to an alternative method, the matrix method [81]. The method estimates the number of multi-jet background events in the signal region based on loose and tight lepton isolation definitions, the latter selection being a subset of the former. The number of multi-jet events $N^\text {tight}_\text {fake}$ passing the tight (signal) isolation requirements can be expressed as:

$$\begin{aligned} N^\text {tight}_\text {fake} = \frac{\epsilon _\text {fake}}{\epsilon _\text {real} - \epsilon _\text {fake}} \cdot (N^\text {loose} \epsilon _\text {real} - N^\text {tight})\,, \end{aligned}$$

where $\epsilon _\text {real}$ and $\epsilon _\text {fake}$ are the efficiencies for real and fake loose leptons being selected as tight leptons, $N^\text {loose}$ is the number of selected events in the loose sample, and $N^\text {tight}$ is the number of selected events in the signal sample. By comparing the two methods, the uncertainty on the fraction of multi-jet events is estimated to be 17 %. The shape uncertainty is constructed by comparing the neural-network output distributions of the jet-lepton and anti-muon samples with the distributions obtained using the matrix method.

### Monte Carlo generators

Systematic effects from the modelling of the signal and background processes are taken into account by comparing different generator models and varying the parameters of the event generation. The effect of parton-shower modelling for the top-quark processes is tested by comparing two Powheg samples interfaced to Herwig and Pythia, respectively. There are also differences associated with the way in which double-counted events in the NLO corrections and the parton showers are removed. These are estimated by comparing samples produced with the MC@NLO method and the Powheg method.

The difference between the top-quark mass used in the simulations and the measured value has negligible effect on the results.

For the single top-quark processes, variations of initial- and final-state radiation (ISR and FSR) together with variations of the hard-process scale are studied. The uncertainty is estimated using events generated with Powheg interfaced to Pythia. Factorisation and renormalisation scales are varied independently by factors of 0.5 and 2.0, while the scale of the parton shower is varied consistently with the renormalisation scale using specialised Perugia 2012 tunes [44]. The uncertainty on the amounts of ISR and FSR in the simulated $t\bar{t}$ sample is assessed using Alpgen samples, showered with Pythia, with varied amounts of initial- and final-state radiation, which are compatible with the measurements of additional jet activity in $t\bar{t}$ events [86].

The effect of applying the *W*-boson $p_{\text {T}}$ reweighting was studied and found to have negligible impact on the shape of the neural-network output distribution and the measured cross-section. Hence no systematic uncertainty due to this was assigned.

Finally, an uncertainty is included to account for statistical effects from the limited size of the MC samples.

### Parton distribution functions

Systematic uncertainties related to the parton distribution functions are taken into account for all samples using simulated events. The events are reweighted according to each of the PDF uncertainty eigenvectors or replicas and the uncertainty is calculated following the recommendation of the respective PDF group [73]. The final PDF uncertainty is given by the envelope of the estimated uncertainties for the CT10 PDF set, the MSTW2008 PDF set and the NNPDF 2.3 PDF set.

### Theoretical cross-section normalisation

The theoretical cross-sections and their uncertainties are given in Sect. 4.2 for each background process. Since the single top-quark *t*-, *Wt*-, and *s*-channel processes are grouped together in the statistical analysis, their uncertainties are added in proportion to their relative fractions, leading to a combined uncertainty of 10 %.

A cross-section uncertainty of 4 % is assigned for the *W* / *Z*+(0 jet) process, while ALPGEN parameter variations of the factorisation and renormalisation scale and the matching parameter consistent with experimental data yield an uncertainty on the cross-section ratio of 24 %. For $W$+HF production, a conservatively estimated uncertainty on the HF fraction of 50 % is added. This uncertainty is also applied to the combined *Z*+jets and diboson background.

### Luminosity

The uncertainty on the measured luminosity is estimated to be 2.8 %. It is derived from beam-separation scans performed in November 2012, following the same methodology as that detailed in Ref. [87].

## Results

In order to estimate the signal content of the selected sample, a binned maximum-likelihood fit to the complete neural-network output distributions in the signal region is performed. Including all bins of the neural-network output distributions in the fit has the advantage of making maximal use of all signal events remaining after the event selection, and, in addition, allows the background acceptances to be constrained by the data.

The signal rates, the rate of the single top-quark and $t\bar{t}$ background and the rate of the $W$+HF background are fitted simultaneously. The event yields of the multi-jet background, the $W$+LF and the combined *Z*+jets/diboson background are not allowed to vary in the fit, but instead are fixed to the estimates given in Table 1.

No significant rate of FCNC single top-quark production is observed. An upper limit is set using hypothesis tests. The compatibility of the data with the signal hypothesis, which depends on the coupling constants, and the background hypothesis is evaluated by performing a frequentist hypothesis test based on pseudo-experiments, corresponding to an integrated luminosity of 20.3 fb$^-$ $^1$. Two hypotheses are compared: the null hypothesis, $H_0$, and the signal hypothesis, $H_1$, which includes FCNC single top-quark production. For both scenarios, ensemble tests, i.e. large sets of pseudo-experiments, are performed. Systematic uncertainties are included in the pseudo-experiments using variations of the signal acceptance, the background acceptances and the shape of the neural-network output distribution due to all sources of uncertainty described in the previous section.

To distinguish between the two hypotheses, the so-called *Q* value is used as a test statistic. It is defined as the ratio of the likelihood function *L*, evaluated for the different hypotheses:

2 $$\begin{aligned} Q = -2 \ln \left( \frac{L\left( \beta ^\text {FCNC} = 1 \right) }{ L\left( \beta ^\text {FCNC} = 0 \right) } \right) , \end{aligned}$$

where $\beta ^\text {FCNC}$ is the scale factor for the number of events expected from the signal process for an assumed production cross-section. Systematic uncertainties are included by varying the predicted number of events for the signal and all background processes in the pseudo-experiments.

The $\text {CL}_{s}$ method [88] is used to derive confidence levels ($\text {CL}$) for a certain value of $Q^{\text {obs}}$ and $Q^{\text {exp}}$. A particular signal hypothesis $H_1$, determined by given coupling constants $\kappa _{ugt}/\Lambda$ and $\kappa _{cgt}/\Lambda$, is excluded at the 95 % $\text {CL}$ if a $\text {CL}_{s}< 0.05$ is found. The observed 95 % $\text {CL}$ upper limit on the anomalous FCNC single top-quark production cross-section multiplied by the $t \rightarrow Wb$ branching fraction, including all uncertainties, is 3.4 pb, while the expected upper limit is ${2.9^{+1.9}_{-1.2}}$pb.

**Fig. 9:**
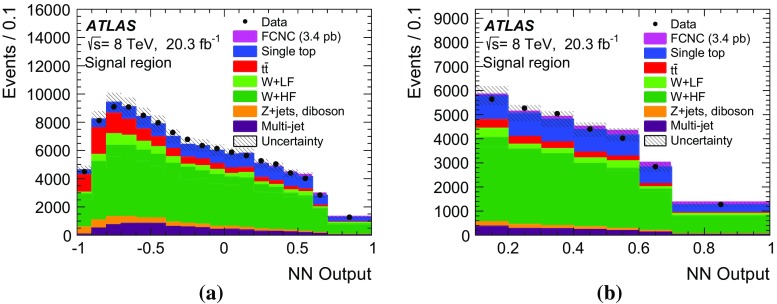
**a** Neural-network output distribution in the signal region and **b** in the signal region with neural network output above 0.1. In both figures the signal contribution scaled to the observed upper limit is shown. The *hatched band* indicates the total posterior uncertainty as obtained from the limit calculation

To visualise the observed upper limit in the neural-network output distribution, the FCNC signal process scaled to 3.4 pb stacked on top of all background processes is shown in Fig. 9.

The total uncertainty is dominated by the jet energy resolution uncertainty, the modelling of $E_{\text {T}}^{\text {miss}}$ and the uncertainty on the normalisation and the modelling of the multi-jet background. A summary of all considered sources and their impact on the expected upper limit is shown in Table 3.

**Table 3:** The effect of a single systematic uncertainty in addition to the cross-section normalisation and MC statistical uncertainties alone (top row) on the expected 95 % $\text {CL}$ upper limits on the anomalous FCNC single top-quark production $qg\rightarrow t \rightarrow b\ell \nu$. The relative change quoted in the third column is with respect to the expected limit with normalisation and MC statistical uncertainties only

Source	Expected 95 % $\text {CL}$ upper limit (pb)	Change in the upper limit (%)
Normalisation and MC statistics	1.5	–
Multi-jets normalisation and modelling	1.8	25
Luminosity	1.5	5
Lepton identification	1.5	3
Electron energy scale	1.6	8
Electron energy resolution	1.5	4
Muon momentum scale	1.5	1
Muon momentum resolution	1.5	5
Jet energy scale	1.6	8
Jet energy resolution	1.9	32
Jet reconstruction efficiency	1.5	4
Jet vertex fraction scale	1.5	3
*b*-tagging efficiency	1.5	3
*c*-tagging efficiency	1.5	4
Mistag acceptance	1.5	2
$E_{\text {T}}^{\text {miss}}$ modelling	1.9	34
PDF	1.5	5
Scale variations	1.5	2
MC generator (NLO subtraction method)	1.6	8
Parton shower modelling	1.5	5
All systematic uncertainties	2.9	–

**Fig. 10:**
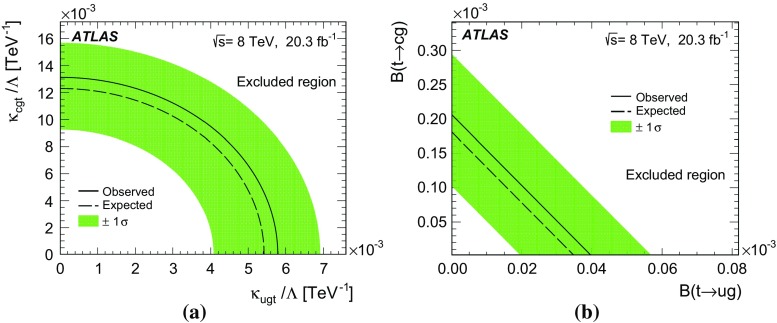
**a** Upper limit on the coupling constants $\kappa _{ugt}$ and $\kappa _{cgt}$ and **b** on the branching fractions $\mathcal {B}(t \rightarrow ug)$ and $\mathcal {B}(t \rightarrow cg)$. The *shaded band* shows the one standard deviation variation of the expected limit

Using the NLO predictions for the FCNC single top-quark production cross-section [89, 90] and assuming $\mathcal {B}(t \rightarrow Wb) = 1$, the upper limit on the cross-section can be interpreted as a limit on the coupling constants divided by the scale of new physics: $\kappa _{ugt}/\Lambda < {5.8 \times 10^{-3} \,\mathrm{TeV}}$ assuming $\kappa _{cgt}/\Lambda = 0$, and $\kappa _{cgt}/\Lambda < {13 \times 10^{-3}\, \mathrm{TeV}}$ assuming $\kappa _{ugt}/\Lambda = 0$. Distributions of the upper limits on the coupling constants for combinations of *cgt* and *ugt* channels are shown in Fig. 10a.

Limits on the coupling constants can also be interpreted as limits on the branching fractions using $\mathcal {B}(t \rightarrow qg) = \mathcal {C} \left( \kappa _{qgt} / \Lambda \right) ^{2}$, where $\mathcal {C}$ is calculated at NLO [91]. Upper limits on the branching fractions $\mathcal {B}(t \rightarrow ug) < 4.0 \times 10^{-5}$, assuming $\mathcal {B}(t \rightarrow cg)=0$ and $\mathcal {B}(t \rightarrow cg) < {20 \times 10^{-5}}$, assuming $\mathcal {B}(t \rightarrow ug)=0$, are derived and presented in Fig. 10b.

## Conclusion

A search for anomalous single top-quark production via strong flavour-changing neutral currents in *pp* collisions at the LHC is performed. Data collected by the ATLAS experiment in 2012 at a centre-of-mass energy $\sqrt{s} = {8}\mathrm{TeV}$, and corresponding to an integrated luminosity of 20.3 fb$^{-1}$ are used. Candidate events for which a *u*- or *c*-quark interacts with a gluon to produce a single top quark are selected. To discriminate between signal and background processes, a multivariate technique using a neural network is applied. The final statistical analysis is performed using a frequentist technique. As no signal is seen in the observed output distribution, an upper limit on the production cross-section is set. The expected 95 % $\text {CL}$ limit on the production cross-section multiplied by the $t \rightarrow b W$ branching fraction is $\sigma _{qg \rightarrow t} \times \mathcal {B}(t \rightarrow bW)< {2.9}\,\mathrm{pb}$ and the observed 95 % $\text {CL}$limit is $\sigma _{qg \rightarrow t} \times \mathcal {B}(t \rightarrow Wb)< {3.4}\,\mathrm{pb}$. Upper limits on the coupling constants divided by the scale of new physics $\kappa _{ugt}/\Lambda < {5.8\times 10^{-3}}\,\mathrm{TeV}$ and $\kappa _{cgt}/\Lambda < {13 \times 10^{-3} \,\mathrm{TeV}}$ and on the branching fractions $\mathcal {B}(t \rightarrow ug) < {4.0\times 10^{-5}}$ and $\mathcal {B}(t \rightarrow cg) < {20 \times 10^{-5}}$ are derived from the observed limit. These are the most stringent limits published to date.

## Footnotes

1 Charge conjugate production and decay modes are implied throughout this paper.

2 ATLAS uses a right-handed coordinate system with its origin at the nominal interaction point in the centre of the detector and the *z*-axis is along the beam direction; the *x*-axis points towards the centre of the LHC ring and the *y*-axis points upwards. The pseudorapidity $\eta$ is defined as $\eta =-\ln [\tan (\theta /2)]$, where the polar angle $\theta$ is measured with respect to the *z*-axis. The azimuthal angle, $\phi$, is measured with respect to the *x*-axis. Transverse momentum and energy are defined as $p_{\text {T}}= p\sin \theta$ and $E_{\text {T}}= E\sin \theta$, respectively. The $\Delta R$ distance in ($\eta$,$\phi$) space is defined as $\Delta R=\sqrt{(\Delta \eta )^2+(\Delta \phi )^2}$.

3 A right-handed top quark would give rise to different angular distributions and hence be easier to separate from SM production.

4 The primary vertex is defined as the vertex with the largest $\sum p_{\text {T}}^2$ of the associated tracks.

5 The *W*-boson transverse mass is defined as: $m_{\text {T}}(W)= \sqrt{ 2 \left( p_{\text {T}}(\ell ) E_{\text {T}}^{\text {miss}}- \mathbf {p}_{\text {T}}(\ell ) \cdot \mathbf {E}_{\text {T}}^{\text {miss}}\right) }$, where $\mathbf {p}_{\text {T}}(\ell )$ denotes the transverse momentum of the lepton and $p_{\text {T}}(\ell ) = |\mathbf {p}_{\text {T}}(\ell )|$.

6 The ranking is done according to the correlation to the output.
